# Structural dynamics of IDR interactions in human SFPQ and implications for liquid–liquid phase separation

**DOI:** 10.1107/S2059798325005303

**Published:** 2025-06-27

**Authors:** Heidar J. Koning, Valerie Lai, Ashish Sethi, Shatabdi Chakraborty, Ching-Seng Ang, Archa H. Fox, Anthony P. Duff, Andrew E. Whitten, Andrew C. Marshall, Charles S. Bond

**Affiliations:** a School of Molecular Sciences The University of Western Australia Crawley WA6009 Australia; b Australian Nuclear Science and Technology Organisation, The Australian Synchrotron 800 Blackburn Road Clayton VIC3168 Australia; c The Bio21 Molecular Science and Biotechnology Institute University of Melbourne Parkville VIC3010 Australia; d Department of Biochemistry and Pharmacology University of Melbourne Parkville VIC3010 Australia; e School of Human Sciences The University of Western Australia Crawley WA6009 Australia; f ANSTO, New Illawarra Road, Lucas Heights, NSW2234, Australia; McGill University, Canada

**Keywords:** phase separation, disorder, flexibility, dimers, DBHS

## Abstract

The proteins SFPQ (splicing factor proline- and glutamine-rich) and NONO (non-POU domain-containing octamer-binding protein) are members of the *Drosophila* behaviour/human splicing (DBHS) protein family, sharing 76% sequence identity in their conserved DBHS domain. These proteins are critical for elements of pre- and post-transcriptional regulation in mammals and are primarily located in paraspeckles: ribonucleoprotein bodies templated by NEAT1 long noncoding RNA. Regions that are structured and predicted to be disordered (IDRs) in DBHS proteins facilitate various interactions, including dimerization, polymerization, nucleic acid binding and liquid–liquid phase separation, all of which have consequences for cell health, the pathology of some neurological diseases and cancer. To date, very limited structural work has been carried out on characterizing the IDRs of the DBHS proteins, largely due to their predicted disordered nature and the fact that this is often a bottleneck for conventional structural techniques. This is a problem worth addressing, as the IDRs have been shown to be critical to the material state of the protein as well as its function. In this study, we used small-angle X-ray scattering (SAXS) and small-angle neutron scattering (SANS), together with lysine cross-linking mass spectrometry (XL-MS), to investigate the regions of SFPQ flanking the structured DBHS domain and the possibility of dimer partner exchange of full-length proteins. Our results demonstrate experimentally that the N- and C-terminal regions on either side of the folded DBHS domain are long, disordered and flexible in solution. Realistic modelling of disordered chains to fit the scattering data and the compaction of the different protein variants suggests that it is physically possible for the IDRs to be close enough to interact. The mass-spectrometry data additionally indicate that the C-terminal IDR can potentially interact with the folded DBHS domain and also shares some conformational space with the N-terminal IDR. Our small-angle neutron scattering (SANS) experiments reveal that full-length SFPQ is capable of swapping dimer partners with itself, which has implications for our understanding of the combinatorial dimerization of DBHS proteins within cells. Our study provides insight into possible interactions between different IDRs either in *cis* or in *trans* and how these may relate to protein function, and the possible impact of mutations in these regions. The dynamic dimer partner exchange of a full-length protein inferred from this study is a phenomenon that is integral to the function of DBHS proteins, allowing changes in gene-regulatory activity by altering levels of the various heterodimers or homodimers.

## Abstract 2

SFPQ and NONO are DBHS-family proteins that are essential for transcriptional regulation and the assembly of paraspeckles, which are subnuclear structures built on the long noncoding RNA NEAT1. Through SAXS, SANS and XL-MS analyses, we reveal that the disordered regions of SFPQ surrounding its DBHS domain enable flexible interactions and that the full-length proteins are capable of dimer partner exchange, highlighting how these dynamics may contribute to phase separation and impact disease processes.

## 1. Introduction

### 1.1. Biological functions of DBHS proteins

SFPQ and NONO are functionally diverse proteins that are ubiquitously present in mammalian nucleic acid-processing pathways, as reviewed by Knott *et al.* (2016 ▸). The known functions of SFPQ/NONO involve a role in almost every step of nucleic acid processing in mammalian cells, where they are involved in sequestration, co-repression/co-activation of transcription, RNA export, transport and retention, elongation/termination, co-transcriptional processing and the formation of subnuclear bodies (Knott *et al.*, 2016 ▸). SFPQ is so involved in cellular function that studies have shown that it is a critical protein for life in mammals, with embryo-level knockouts causing organism death (Takeuchi *et al.*, 2018 ▸). Interestingly, SFPQ and NONO are critical proteins for the assembly of the core region of paraspeckles: dynamic phase-separated nuclear condensates that are templated by approximately 50 molecules of the 23 kbp long noncoding RNA NEAT1 and are known to sequester, regulate and organize multiple types of RNA and proteins via liquid–liquid phase separation (LLPS) and extensive multivalency (Fox *et al.*, 2018 ▸; West *et al.*, 2016 ▸). SFPQ is also an emerging actor in neurodegenerative disease research due to its critical role in the development and regulation of neurons at multiple tiers of nucleic acid processing such as transcription, splicing, axonal RNA transport and stress-granule formation (Lim *et al.*, 2020 ▸). The imbalanced nucleocytoplasmic distribution of SFPQ is reportedly a factor in the neurodegenerative diseases amyotrophic lateral sclerosis (ALS), frontotemporal lobar degeneration (FTLD) and Alzheimer’s disease (AD) (Lim *et al.*, 2020 ▸).

### 1.2. General architecture and behaviour of DBHS proteins

**Figure 1:**
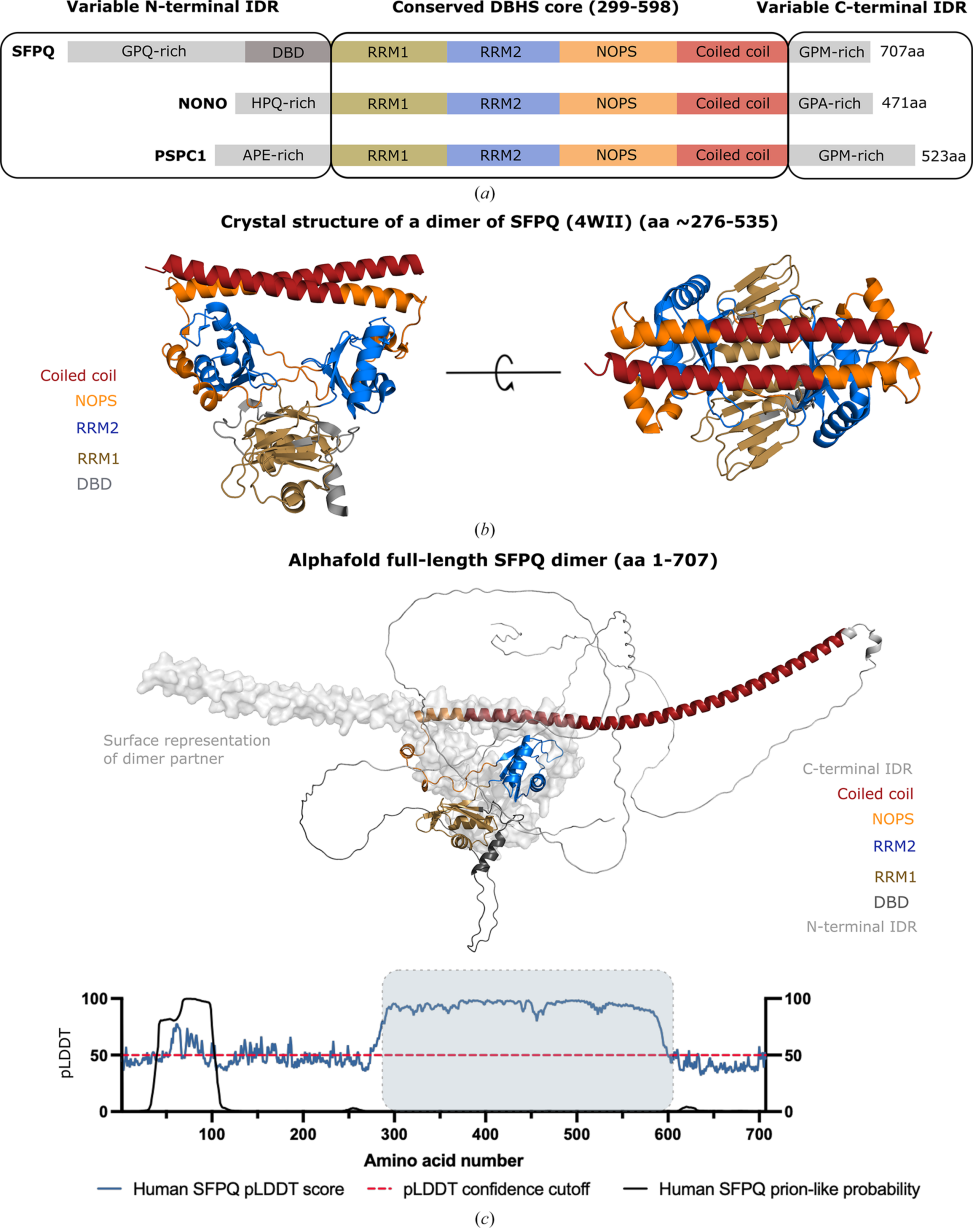
The DBHS family, dimerization and disorder. (*a*) The domain map of the DBHS family indicates the conserved central DBHS region coloured by domain (gold for RRM1, blue for RRM2, orange for NOPS and red for the coiled-coil domain). The different IDRs and the DBD are coloured grey. (*b*) Side view and top view of the structure of an SFPQ homodimer (PDB entry 4wii; Lee *et al.*, 2015 ▸). The protein variant was truncated to remove the extended coiled-coil domain and disordered regions. This structure has been coloured according to the domain map in (*a*). (*c*) Predicted *AlphaFold*2 (Mirdita *et al.*, 2022 ▸) structure of human full-length SFPQ coloured according to the domain map in (*a*). One monomer in the dimer is shown as a cartoon representation and the other as a surface representation without IDRs for simplicity. Light grey regions are the N- and C-terminal IDRs represented as ‘barbed wire’ by *AlphaFold*. Below the predicted structure *AlphaFold* pLDDT and PLAAC prion-like probability (Lancaster *et al.*, 2014 ▸) scores for human SFPQ as a function of amino-acid number are shown. A pLDDT score above ∼50 is a good indicator of structure and a score below ∼50 is indicative of disorder. A PLAAC score approaching 1 (100) is indicative of prion-like characteristics/sequence.

SFPQ and NONO, along with PSPC1, are the mammalian paralogs of the *Drosophila* behaviour/human splicing (DBHS) protein family and share 76% sequence identity in their conserved DBHS domain (Knott *et al.*, 2016 ▸; Fig. 1 ▸*a*).

The DBHS domain is a structured 320-amino-acid conserved core region which contains two RNA-recognition motifs (RRMs), a NonA paraspeckle domain (NOPS) and a coiled-coil domain (Fig. 1 ▸*a*; Knott *et al.*, 2016 ▸). SFPQ, NONO and PSPC1 form obligate dimers, with the core DBHS region being responsible for directing homodimerization and heterodimerization via an extensive network of stable interactions between monomers (Figs. 1 ▸*b* and 1 ▸*c*). The analysis by Passon *et al.* (2012 ▸) of the PSPC1–NONO dimer revealed that ∼25% of the solvent-accessible space of each monomer was buried as a result of dimerization, while analysis by Lee *et al.* (2015 ▸) of the interaction energy of the dimerization interface in a SFPQ homodimer revealed a very stable, high-affinity interaction (Δ^i^*G* = −42.7 kcal mol^−1^, *p*-value = 0.05; Figs. 1 ▸*b* and 1 ▸*c*). The interaction is well preserved across the family, with crystal structures of all six dimeric permutations having been previously determined and analysed [Huang *et al.*, 2018 ▸ (PDB entry 5wpa); Knott *et al.*, 2022 ▸ (PDB entries 5ifn and 5ifm); Lee *et al.*, 2015 ▸ (PDB entries 4wii, 4wik and 4wij); Passon *et al.*, 2012 ▸ (PDB entry 3sde); Lee *et al.*, 2022 ▸ (PDB entry 7lrq); Schell *et al.*, 2022 ▸ (PDB entry 7pu5)]. An additional intermediate part of the DBHS conserved region is responsible for functional aggregation via a coiled-coil-forming interface, which plays a role in the cooperative binding of larger nucleic acids (Figs. 1 ▸*b* and 1 ▸*c*; Koning *et al.*, 2025 ▸; Lee *et al.*, 2015 ▸)

Despite strong evidence outlining the general functions of DBHS proteins and an apparent hierarchy of dimerization configurations (Huang *et al.*, 2018 ▸, Knott *et al.*, 2022 ▸; Lee *et al.*, 2022 ▸), the role of the various dimers is poorly understood. The direct involvement of the DBHS region in nucleic acid interaction (Knott *et al.*, 2022 ▸; Lee *et al.*, 2015 ▸; Vickers & Crooke, 2016 ▸; Wang *et al.*, 2022 ▸) suggests a potential biological role for this combinatorial expansion. Of note, the direct exchange of partners has been demonstrated *in vitro* (Lee *et al.*, 2022 ▸) between SFPQ and NONO homodimers truncated to contain only the dimerization domain, resulting in a population of SFPQ heterodimers. Mechanistically, how partner swapping without cofactors occurs is currently unknown, and is remarkable considering the interaction energies of the various DBHS dimer interfaces. Lee *et al.* (2022 ▸) provided some clues through the identification of certain features such as a helix in the NOPS domain and the relative position of RRM1 in both molecules, which differed across various dimers, suggesting that instability or flexibility of certain stabilizing interactions may be involved in partner swapping or preferential dimerization. To date, direct partner exchange of full-length proteins has not been shown and has implications for understanding the roles of dimers within cells.

### 1.3. Intrinsically disordered regions in DBHS proteins and regulation of liquid–liquid phase separation

Outside of the DBHS domain, the three human paralogs are flanked by extensive regions which vary substantially in sequence and are predicted to be intrinsically disordered (IDR, intrinsically disordered region; Fig. 1 ▸*c*). Previously, this disorder had not been shown experimentally, but becomes apparent when using many sequence-structure prediction tools such as the *AlphaFold* pairwise local distance difference test (pLDDT; Fig. 1 ▸*c*, bottom; Mirdita *et al.*, 2022 ▸) and the *RIDAO* (*Rapid Prediction and analysis of Protein Disorder Online*) suite of disorder-prediction tools (Dayhoff & Uversky, 2022 ▸). Together, these tools and others such as *IUPred*2*A* (Marshall *et al.*, 2023 ▸) indicate that these regions are highly likely to be flexible and disordered. Interestingly, these regions have also been shown, in the case of SFPQ (Marshall *et al.*, 2023 ▸), or predicted (Supplementary Figs. S1 and S2) to be capable of driving liquid–liquid phase separation (LLPS). Recently, Marshall *et al.* (2023 ▸) added further nuance to this idea by examining the contributions of the two predicted flanking IDRs of SFPQ towards LLPS. The predicted IDRs of SFPQ were shown experimentally to be directly involved in LLPS, with the C-terminal IDR driving phase separation and the N-terminal IDR attenuating phase separation (Marshall *et al.*, 2023 ▸). Marshall *et al.* (2023 ▸) proposed a possible direct regulatory interaction between the IDRs of SFPQ in the context of individual dimers for the purpose of modulating condensate formation in the nucleus.

Structural studies of LLPS proteins are often challenging as their high degrees of disorder, number of dynamic conformations, solubility and capacity for oligomerization and phase separation can make crystallization or studies with electron microscopy difficult or impossible (Martin, Hopkins *et al.*, 2021 ▸). For this reason, despite the exhaustive characterization of the structured DBHS domain, the structural details of the predicted IDRs of SFPQ and whether a direct intradimer interaction between the IDRs is possible in solution have yet to be described experimentally. The potential for combinatorial dimerization and dimer partner exchange of full-length proteins under near-physiological conditions may impact on LLPS: it is possible that nature uses the divergent sequence features of each paralog through combinatorial dimerization to further control DBHS protein LLPS and the material properties of nuclear condensates.

Small-angle scattering using X-rays or neutrons (SAXS or SANS) has emerged as an effective method for studying disordered proteins structurally in solution. In this study, we employ both SAXS and SANS, in conjunction with lysine cross-linking mass spectrometry (XL-MS), to gain insights into the structure and dynamics of the predicted intrinsically disordered regions (IDRs) of SFPQ and to explore the potential for dimer partner exchange of full-length proteins *in vitro*. Firstly, we compared the scattering of a tractable truncate of SFPQ missing the C-terminal IDR (SFPQ1–598) and compared it with the data for the full-length protein (SFPQ1–707). Our solution scattering data demonstrate experimentally that the N- and C-terminal IDRs of SFPQ are long, disordered and flexible in solution. Ensembles of models generated with *EOM* 2.0 (*Ensemble Optimization Method*) suggest that a direct interaction between the IDRs as hypothesized by Marshall *et al.* (2023 ▸) is possible. Such an interaction may explain some degree of compaction seen in the ensemble that fits the scattering data relative to the initial pool of search models. The cross-linking mass-spectrometry data also encouragingly show that the distal ends of the C-terminal IDR can make points of contact with the folded domain and that both IDRs can come into close proximity to one another in solution. We additionally demonstrate that full-length protiated SFPQ is capable of swapping dimer partners in solution with other molecules of deuterated SFPQ and that it is possible to capture scattering data of the full-length protein as a monomer in place of a dimer using contrast-matching small-angle neutron scattering (SANS).

In this study, we show the first structural description of the IDRs of SFPQ, and their potential dynamics in solution, as well as the capability of full-length SFPQ dimers to exchange partners with each other in a stable manner *in vitro*. These findings are biologically relevant as the IDRs directly control the material state of SFPQ and are either directly or indirectly involved in all of the biological functions of the protein. Additionally, partner swapping between full-length DBHS proteins is likely to allow multiple possible interactions between IDRs of different dimers and the modulation of phase properties via unique combinations of dimers within condensates. Together, these factors are important for paraspeckle formation, disease pathology and the several functions that SFPQ carries out that are critical to mammalian life.

## 2. Materials and methods

### 2.1. Protein expression of deuterated and protiated SFPQ

The plasmids (i) pET-mEGFP-SFPQ (full-length) and (ii) pET-mEGFP-SFPQ (1–598) were transformed into Invitrogen OneShot BL21 Star (DE3) cells separately. The proteins were expressed using RTF bioreactors according to the method of Duff *et al.* (2015 ▸). In all cases, the medium was composed of ModC1, 78.1% D_2_O and 40 g l^−1^^1^H-glycerol. 78.1% D_2_O was chosen, using empirical data on past protein deuteration runs, to achieve a neutron scattering length density match point equivalent to 95% D_2_O. The proteins were induced with 0.5 m*M* isopropyl β-d-1-thiogalactopyranoside (IPTG) at OD_600 nm_ values of (i) 12.44 and (ii) 12.18 for subsequent expression at 20°C. The cells were harvested directly after exhaustion of the carbon source, as shown by a small rise in pH above the setpoint of 6.2. Deuteration levels were determined by MS (partial trypsin digest MALDI-TOF). In some cases the MS spectra were low quality and a precise deuteration level was unable to be achieved; however, all results are consistent with a deuteration level of 61.5 ± 0.5%. A consistent deuteration level was expected due to the medium and growth characteristics being the same in both cases.

For the production of unlabelled biomass the steps were the same as above, but no D_2_O was used to ensure the expression of protiated versions of full-length SFPQ and SFPQ1–598. The proteins were induced with 0.5 m*M* IPTG at OD_600 nm_ values of (i) 13.24 and (ii) 12.15 for subsequent expression at 20°C. The cells were harvested directly after exhaustion of the carbon source, as indicated by a small rise in pH above the setpoint of 6.2. In all cases the medium also contained 40 µg ml^−1^ kanamycin to maintain plasmid selection.

### 2.2. Protein purification of deuterated and protiated full-length SFPQ and SFPQ1–598

For the purification of all of the variants of SFPQ used in this study, the purification buffers from Marshall *et al.* (2023 ▸) were used. Lysis was carried out in the buffer 1 *M* KCl, 5% glycerol, 10 m*M* imidazole, 50 m*M* Tris–HCl, 250 m*M*l-arginine, 1 m*M* PMSF; addition of PMSF to all steps was optional for the purification of SFPQ1–598 but was necessary for full-length SFPQ. Frozen biomass was chipped from the container into a sterile and clean Schott bottle. The Schott bottle was then filled to a final volume 24 times that of the biomass (*i.e.* 10 g of biomass resuspended in 240 ml solution). This was performed using a mixture of 50 ml BugBuster 10× Protein Extraction Reagent (Merck) at a 1/10 final volume and a 9/10 final volume of lysis buffer supplemented with DNase I (Merck) at 50 µg ml^−1^, two cOmplete Mini EDTA-free protease-inhibitor cocktail tablets (for this volume) and lysozyme to a final concentration of 0.2 mg ml^−1^. The sample mixture was stirred at room temperature using a magnetic stirrer for ∼1 h until adequate resuspension/dissolution of the biomass into solution.

Lysates were then clarified by centrifugation and filtered using Whatman 0.4 µm filters and a vacuum degassing setup to reduce sample viscosity. Following filtration, the lysate was loaded using a peristaltic pump (Bio-Rad) onto 5 ml nickel-affinity columns (GE Healthcare) pre-equilibrated with ten column volumes of water and ten column volumes of binding buffer (1 *M* KCl, 5% glycerol, 10 m*M* imidazole, 50 m*M* Tris–HCl, 250 m*M*l-arginine, 1 m*M* PMSF pH 7.4). The column was then washed with ten column volumes of binding buffer, followed by 5–10 column volumes of binding buffer spiked with 13% elution buffer (binding buffer with 250 m*M* imidazole) to remove further contaminants (five column volumes were sufficient for SFPQ1–598). His-tagged protein was then eluted in ∼1–1.5 column volumes of nickel elution buffer. To remove the GFP tag, the eluted protein was subjected to an overnight digest with Tobacco etch virus protease at a 1:25 mass ratio. This digest was dialysed overnight at room temperature with a magnetic stirrer in ∼1 l nickel binding buffer supplemented with DTT to a final concentration of 1 m*M*.

Following this, the sample was recovered from the tubing, filtered using a 0.4 µm syringe filter and then flowed over a 5 ml nickel-affinity column (pre-equilibrated in binding buffer) to remove the TEV protease and residual GFP. The sample was further pushed through the column with binding buffer containing 5% elution buffer to remove any nonspecific interactions between SFPQ and the nickel resin (*i.e.* 5 ml was loaded onto the column and ∼10 ml was recovered). The sample was then purified by loading the eluate onto a Superdex 200 16/60 size-exclusion column pre-equilibrated in storage buffer (0.5 *M* KCl, 5% glycerol, 20 m*M* HEPES, 1 m*M* DTT pH 7.4). Elution peaks were monitored using the absorbance at 280 nm. The eluted protein was analysed with SDS–PAGE, flash-frozen with liquid nitrogen and stored at −80°C until further use.

### 2.3. Sequence analysis and structure prediction

Sequence analysis of SFPQ was performed using *AlphaFold* pLDDT scores retrieved from *ColabFold* (Mirdita *et al.*, 2022 ▸) to test for regions of predicted structure outside of the DBHS domain. Phase-separating regions for the DBHS proteins were predicted using the *FuzDrop* tool (Hatos *et al.*, 2022 ▸).

Protein amino-acid composition analyses were performed using custom *R* scripts available at https://github.com/acmarshall88/AA-CounteR. To calculate the ‘enrichment’ of each of the 20 naturally occurring amino acids in a protein sequence of interest, the proportion of each amino acid was calculated and divided by its proportion within the entire human proteome (UniProt ID UP000005640_9606; contains one protein sequence per gene). Grey bars indicate infinite depletion (*i.e.* that amino-acid type is absent from the sequence). To visualize the occurrence of amino acids that are particularly enriched at any point along each DBHS protein sequence, the proportion of each amino acid was calculated in a sliding window of pre-defined width (*i.e.* 30 amino acids) across each protein sequence.

### 2.4. Small-angle X-ray scattering

#### 2.4.1. Measurements, data reduction and analysis

Small-angle X-ray scattering (SAXS) data for all SFPQ/NONO constructs were collected on the SAXS/WAXS beamline at the Australian Synchrotron using an inline SEC-SAXS (size-exclusion chromatography–small-angle X-ray scattering) co-flow setup (Kirby *et al.*, 2016 ▸; Ryan *et al.*, 2018 ▸). Data were all collected using a buffer consisting of 500 m*M* KNO_3_, 20 m*M* HEPES pH 7.4, 5% glycerol, 1 m*M* DTT. To analyse the effect of a low-salt buffer on SFPQ1–598, the protein was concentrated in its initial storage buffer and dialysed overnight into 150 m*M* KCl, 20 m*M* HEPES pH 7.4, 5% glycerol, 5 m*M* MgCl_2_, 1 m*M* DTT. All samples were analysed on a pre-equilibrated Superdex 200 5/150 column (GE Healthcare) with UV absorbance at 260 and 280 nm monitored alongside X-ray scattering. Data reduction was carried out using *SCATTERBRAIN* 2.82 (software for acquiring, processing and viewing SAXS/WAXS data at the Australian Synchrotron; Trewhella *et al.*, 2017 ▸) and corrected for solvent scattering and sample transmission. As discussed by Trewhella *et al.* (2017 ▸), *SCATTERBRAIN* outputs the uncertainty of intensity measurements as 2σ. For the analysis in this paper, these uncertainties were transformed to σ for all data sets such that all metrics used for analysis in programs and for comparing models to experimental data had conventional interpretations.

Data processing and analysis were performed using the *ATSAS* suite (Petoukhov *et al.*, 2012 ▸). For all SEC-SAXS data, self-consistent, non-protein regions were averaged and taken as solvent scattering with *CHROMIXS*. The sample scattering was then taken as the average of frames with similar *R*_g_ values that were measured as the protein eluted. Guinier analysis and Kratky analysis were performed in *ATSAS* 4.0 (Manalastas-Cantos *et al.*, 2021 ▸). Pair-distance distribution functions *P*(*r*) were generated from the experimental data using *GNOM*/*PRIMUS* (Petoukhov *et al.*, 2012 ▸). As the *P*(*r*) function can be subject to bias and experimental artefacts, together with the fact that there can be inherent uncertainty in *D*_max_ which can be difficult to quantify (Trewhella *et al.*, 2017 ▸), we applied consistent criteria to their derivation. *P*(*r*) functions had simultaneously high TQE (total quality estimate) scores, were able to reach *P*(*r*) = 0 smoothly and without forcing, and displayed no systematic variation in the normalized residual plot between the experiment and the regularized fit. For some functions, we further cross-validated our selection of *D*_max_ with the range of physically plausible values seen in our analysis using *EOM*. In the case of full-length SFPQ, to test for possible artefacts in *P*(*r*) *D*_max_ was varied around the chosen value, different *q*-ranges were chosen for the regularized fit and the *GNOM* regularization parameter (α) was varied. The molecular weights and volumes of the various samples were calculated using the method of Fischer *et al.* (2010 ▸).

### 2.5. Small-angle neutron scattering

#### 2.5.1. Calculation of deuteration level and match-out point using *MULCh*

The neutron scattering length density and contrast of SFPQ were calculated using *MULCh* (version 1.1.1; Whitten *et al.*, 2008 ▸). The full-length sequence of SFPQ was used as input, and the volume of the molecule was estimated from the amino-acid composition. A deuteration level of 62.9% (based on MS results) was used, and it was assumed that 90% of the exchangeable H positions were accessible by the solvent. The buffer composition was taken to be 5%(*v*/*v*) glycerol (C_3_H_8_O_3_; a molar concentration of 0.684 *M* and a molecule volume of 121.4 Å^3^ was assumed), 500 m*M* KCl, 20 m*M* HEPES, 1 m*M* DTT. The contrast-matching condition for SFPQ in these buffer conditions was estimated to contain 99.8% buffer made up in D_2_O with 0.2% buffer made up in H_2_O. This corresponds to solution conditions of 94.8% D_2_O, 0.2% H_2_O, 500 m*M* KCl, 5%(*v*/*v*) glycerol, 20 m*M* HEPES, 1 m*M* DTT. In these solution conditions, the contrast of unlabelled SFPQ was estimated to be −2.84 × 10^10^ cm^−2^.

For a buffer composition of 1.5%(*v*/*v*) glycerol, 150 m*M*KCl, 20 m*M* HEPES, 1 m*M* DTT made up in H_2_O, the contrast of unlabelled SFPQ is estimated to be 2.49 × 10^10^ cm^−2^ and the contrast of labelled SFPQ is estimated to be 5.32 × 10^10^ cm^−2^. For a buffer composition of 0.75%(*v*/*v*) glycerol, 150 m*M* KCl, 20 m*M* HEPES, 0.5 m*M* DTT the contrast-matching condition for SFPQ in these buffer conditions was estimated to contain 95.1% buffer made up in D_2_O with 4.9% buffer made up in H_2_O. This corresponds to solution conditions of 94.3% D_2_O, 4.9% H_2_O, 150 m*M* KCl, 1.5%(*v*/*v*)glycerol, 20 m*M* HEPES, 1 m*M* DTT. In these solution conditions, the contrast of unlabelled SFPQ is estimated to be −2.84 × 10^10^ cm^−2^.

#### 2.5.2. SANS match-out testing

For the SANS experimental setup, a storage buffer (500 m*M* KCl, 5% glycerol, 20 m*M* HEPES, 1 m*M* DTT pH 7.4) and a low-salt buffer (20 m*M* HEPES, 1 m*M* DTT pH 7.4) were used. To determine whether proteins could be successfully matched out, 800 µl full-length dSFPQ (deuterated SFPQ; 1.32 mg ml^−1^ in H_2_O storage buffer) was dialysed in 20 ml storage buffer made up in D_2_O overnight at room temperature. The dialyzer was then transferred into 20 ml fresh storage buffer in D_2_O and dialysed for a further 4 h. The H_2_O in the original sample would then have been diluted by a factor of ∼625 (25 × 25). Thus, the final buffer composition of the sample was 94.84% D_2_O, 0.16% H_2_O, 500 m*M* KCl, 5% glycerol, 20 m*M* HEPES, 1 m*M* DTT pH 7.4. Approximately 600 µl of 1.32 mg ml^−1^ dSFPQ in ∼95% D_2_O buffer was transferred into a 2 mm Hellma (‘Banjo’) cell. SANS data were collected using QUOKKA. SANS data were collected in the same way from dialysis buffer (after the final dialysis step) and used for buffer subtraction.

#### 2.5.3. Attempt at producing bulk condensed-phase SFPQ for SANS and resulting scattering in H_2_O

We attempted to produce a bulk condensed phase via dialysis to a lower salt concentration, but this ultimately failed. However, some of the sample from this still produced dimer scattering. 96 ml full-length dSFPQ (1.32 mg ml^−1^ in H_2_O storage buffer; total mass 127 mg) was mixed with 477 µl hSFPQ (13.3 mg ml^−1^ in H_2_O storage buffer; total mass 6.34 mg) such that the hSFPQ:dSFPQ ratio was 1:20. This was dialysed in 224 ml low-salt buffer overnight at room temperature in a 250 ml measuring cylinder. The KCl and glycerol in the original sample would then have been diluted by a factor of 3.33 (320/96). Thus, the final buffer composition of the sample was 150 m*M* KCl, 1.5% glycerol, 20 m*M* HEPES, 1 m*M* DTT pH 7.4. After dialysis, a mass of white/brown precipitate was observed in place of condensed liquid. This was pelleted via centrifugation at 1500*g* for 40 min (20°C). The supernatant (‘dilute phase’) was then removed. The SFPQ concentration in the supernatant was determined to be 0.63 mg ml^−1^ using the absorbance at 280 nm and an extinction coefficient of 0.346 ml mg^−1^ (*ProtParam*). Assuming that the sample contained a 1:20 ratio of hSFPQ:dSFPQ, the concentration of hSFPQ would be 0.0315 mg ml^−1^. Approximately 600 µl of this sample (0.63 mg ml^−1^ of 1:20 hSFPQ:dSFPQ in 150 m*M* KCl, 1.5% glycerol, 20 m*M* HEPES, 1 m*M* DTT pH 7.4 in H_2_O) was transferred into a 2 mm Hellma (‘Banjo’) cell. SANS data were collected using QUOKKA. SANS data were collected in the same way from dialysis buffer (after the dialysis step) and used for buffer subtraction.

#### 2.5.4. Bulk phase attempt and dimer match-out experiment

The remaining ∼100 ml of supernatant (‘dilute phase’) from the experiment described above which had been stored at room temperature for ∼40 h was passed through a 0.2 µm filter and concentrated using 100k molecular-weight cutoff centrifugal devices (Amicon) at ∼35–40°C until the final total volume was 275 µl. The final protein concentration, determined via absorbance at 280 nm, was 48 mg ml^−1^. Therefore, assuming the sample contained a 1:20 ratio of hSFPQ:dSFPQ, the concentration of hSFPQ was 2.4 mg ml^−1^. The concentrated sample was transparent but slightly brown in colour, possibly suggesting the presence of soluble aggregates. This 275 µl sample was then dialysed in 5225 µl of 150 m*M* KCl, 20 m*M* HEPES, 0.5 m*M* DTT pH 7.4 made up in 100% D_2_O overnight at ∼35°C. The dilution of H_2_O and glycerol in the original sample by a factor of 20 meant that the final buffer composition was 150 m*M* KCl, 0.075% glycerol, 20 m*M* HEPES, 0.5 m*M* DTT pH 7.4 in 95% D_2_O. This was loaded warm into a 1 mm Hellma cell, along with ∼50 µl dialysis buffer to ensure that the cell was filled. Turbidity was observed in the cell upon cooling to room temperature. The cell was placed face-down to allow droplets to collect on the surface of the quartz window. SANS data were collected using QUOKKA at two different camera lengths, 1300 and 8000 mm, for 2 and 3 h, respectively. SANS data were collected in the same way from dialysis buffer (after the dialysis step) and used for buffer subtraction.

#### 2.5.5. SANS data reduction and analysis

The data were reduced in the program *IGOR Pro*, where the two-dimensional data were normalized to a common incident neutron count and corrected for sample transmission, background radiation, empty cell scattering and detector sensitivity. The resulting data were then radially averaged to produce *I*(*q*) versus *q* profiles. Scattering data from the two different sample-to-detector distances were then merged, and buffer scattering data were then subtracted from the protein + buffer data to give the resulting protein scattering profiles. Guinier analysis was performed in *ATSAS* 4.0, with PDDF function analysis performed in *PRIMUS* using *GNOM*. *P*(*r*) functions of the SANS data were compared with the SEC-SAXS full-length SFPQ data for analysis of dimer exchange and the conformational state of full-length SFPQ.

### 2.6. 3D modelling

To model the conformers of full-length SFPQ and SFPQ1–598, a model of an SFPQ homodimer (residues 276–598) was generated using *ColabFold* (Mirdita *et al.*, 2022 ▸). In order to generate flexible ensembles, *EOM* 2.0 (Petoukhov *et al.*, 2012 ▸; Tria *et al.*, 2015 ▸) was used to build residues 1–277 and 601–709 as disordered for full-length SFPQ and just residues 1–277 as disordered for SFPQ1–598. To assess the sampling of conformational space by our structures in solution, the distributions of the selected pool that fit the data and the random initial *RanCh* distribution were compared visually and also numerically using values such as the geometric mean *R*_g_, *R*_flex_ and *R*_sigma_. Reduced χ^2^ values were used to assess the agreement of each ensemble with the experimental data, as well as normalized error-weighted residual plots. To model the SANS data, *DAMMIF* was run with ten repetitions on fast mode. The subsequent averaged *DAMAVER* envelope was compared with the atomic structure of a monomer of SFPQ without the IDRs attached.

### 2.7. Lysine cross-linking mass spectrometry (XL-MS)

For cross-linking mass spectrometry (XL-MS) the methodology was essentially the same as the method used by Sethi *et al.* (2023 ▸); purified full-length SFPQ and SFPQ1–598 protein samples were diluted to 10 and 20 µ*M* for both proteins using storage buffer and mixed with a 100-fold excess of DSSO cross-linker (Kao *et al.*, 2012 ▸) dissolved in dimenthyl sulfoxide (DMSO). Following the termination of the cross-linking reaction, the cross-linked proteins were digested with trypsin. LC-MS/MS was performed using a Fusion Lumos Orbitrap mass spectrometer with a FAIMS Pro source (Thermo Fisher, USA). To find the cross-linked peptides, the MS2CID–MS3HCD (MS2–MS3) workflow was used. Cross-linked peptides were then analysed using the *XlinkX* (Liu *et al.*, 2017 ▸) node-implemented *Proteome Discoverer* 2.3 (Thermo Fisher Scientific). The results and subsequent data were then visualized in *xiVIEW* (Combe *et al.*, 2024 ▸).

## 3. Results

### 3.1. Full-length SFPQ in solution revealed by SEC-SAXS

**Figure 2:**
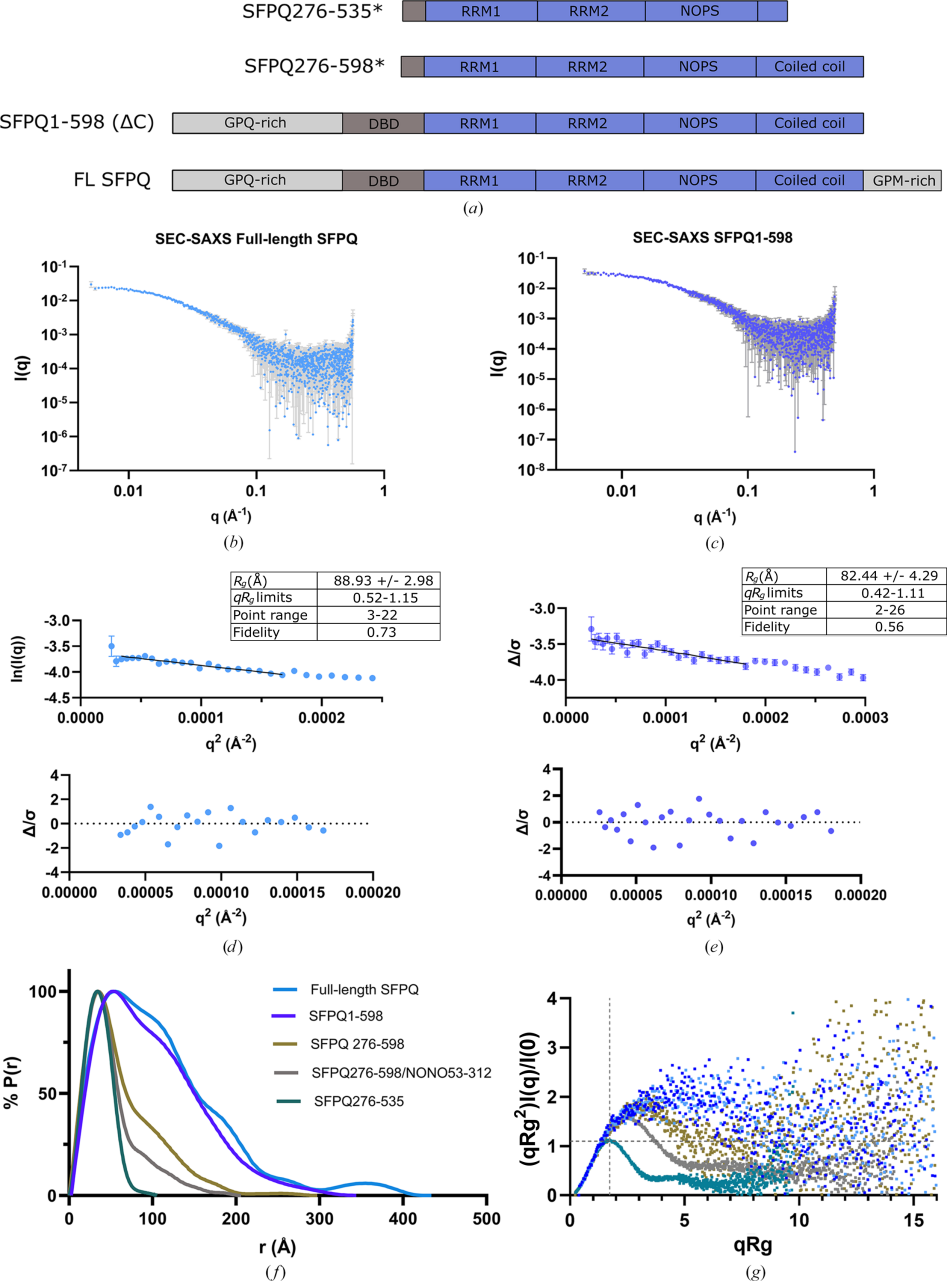
SAXS analysis of SFPQ containing IDRs in high-salt conditions. (*a*) Domain map indicating protein variants that have been analysed via SAXS. An asterisk denotes previously published data or data in the supporting information on variants of SFPQ or NONO. (*b*, *c*) SEC-SAXS scattering for full-length SFPQ and SFPQ1–598, respectively. (*d*, *e*) Guinier analysis for full-length SFPQ and SFPQ1–598, respectively; below, the normalized residuals plots of the Guinier fits. (*f*) Distance distribution functions calculated for all protein variants examined in this study. Functions have been normalized by % *P*(*r*) and error bars have been omitted for simplicity (but can be seen later in the study). (*g*) Dimensionless Kratky plot for all variants used in this study; variants are coloured according to the legend in (*f*).

To investigate the structure of the flanking IDRs of SFPQ in the context of the full-length protein, small-angle X-rayand neutron scattering (SAXS/SANS) experiments were performed on full-length SFPQ (707 residues; includes both IDRs) and on a truncation containing only the N-terminal IDR and the core folded DBHS region (residues 1–598; Fig. 2 ▸*a*). Scattering data from previous studies (Hewage *et al.*, 2019 ▸; Koning *et al.*, 2025 ▸; SASBDB entries SASDFK3 and SASDMG8; Kikhney *et al.*, 2020 ▸) and some unpublished data (Supplementary Fig. S3) of protein variants lacking IDRs were used as a reference for comparison with IDR-containing data sets (Figs. 2 ▸*a*, 2 ▸*f* and 2 ▸*g*). The full set of scattering parameters according to the guidelines set out by Trewhella *et al.* (2023 ▸) are reported in Table 1 ▸.

Initial SEC-SAXS experiments were conducted in high-salt buffers due to the ability of this condition to prevent phase separation, a phenomenon which is not typically useful if one is interested in monodisperse structural information of proteins (Marshall *et al.*, 2023 ▸). However, increasing concentrations of KCl also contain greater amounts of material that can burn onto the capillary under irradiation, potentially causing drifting/sloped baselines in the data. Early attempts at SAXS experiments with full-length DBHS proteins suffered from these problems and were unsuccessful (Yee Seng Chong, personal communication, unpublished data). Both SEC-SAXS experiments on SFPQ variants were performed in potassium nitrate buffers due to the ability of nitrate to function as a powerful radioprotectant through free-radical scavenging (Stachowski *et al.*, 2021 ▸). Additionally, nitrate contributes to a lower scattering background compared with chloride, owing to the lower atomic scattering factors of nitrogen and oxygen compared with chloride.

Both SFPQ experiments in potassium nitrate produced *CHROMIXS* and UV chromatograms indicating the presence of a cleanly separated single peak for both constructs (Supplementary Fig. S4), with UV absorbance ratios at 260:280 nm that were consistent with that of a pure protein solution without any bound nucleic acid (0.62 for full-length SFPQ and 0.688 for SFPQ1–598). A small drift in *R*_g_ across eluted frames can be indicative of flexibility in the sample (Koenigsberg & Heldwein, 2018 ▸) as larger conformers typically elute first. The chromatogram for full-length SFPQ indicated a slight reduction in *R*_g_ across the peak from ∼83 to ∼78 Å (Supplementary Fig. S4). A total of 20 frames across the peak were averaged, where 18 frames varied between 83.4 and 77.1 Å and an additional two frames with *R*_g_ values 74.2 and 75.7 Å were also included. As examined later in the analysis using *EOM*, full-length SFPQ can be modelled by populations of conformers with *R*_g_ values between ∼70 and 100 Å, so a slight variation in *R*_g_ values upon elution is to be expected. This was not the case for the chromatogram of SFPQ1–598, which had some variation in *R*_g_ within the main elution peak, with a small peak surrounded by two more stable plateaus (Supplementary Fig. S4). The first plateau was chosen for averaging, which contained six frames which varied in *R*_g_ from 72.2 to 73.7 Å. Regions prior to protein elution were chosen as the frames for buffer subtraction in *CHROMIXS* (Supplementary Fig. S4).

Each experiment successfully produced monodisperse scattering with log(*I*) versus log(*q*) data sets (Figs. 2 ▸*b* and 2 ▸*c*) having linear fits to the Guinier region (Figs. 2 ▸*d* and 2 ▸*e*) when constrained to *qR*_g_ max = ∼1.1, which can be a necessary limit in accurately determining *R*_g_ for disordered proteins (Zheng & Best, 2018 ▸). The normalized residual plots of each Guinier fit indicated a reasonable degree of variation around the fit and a lack of any curvature (Figs. 2 ▸*d* and 2 ▸*e*, bottom). Comparative *P*(*r*) functions of the different variants from this study indicate the difference in size between an SFPQ dimer containing the structured region and additional variants containing the C- and N-terminal IDRs, which have much larger distributions (Fig. 2 ▸*f*). Interestingly, the distribution for SFPQ1–707 contains a small peak between 300 and 434 Å in the distribution which was initially assumed to be spurious. However, varying *D*_max_ around the tabulated values, fitting different *q*-ranges with *GNOM* and increasing the value of the *GNOM* regularization parameter (α) all allow the peak to persist (Supplementary Fig. S5). Typically, the stability of each distance distribution function with varying α is an additional criterion that can be used to assess the correctness of the solution (Svergun, 1992 ▸). Additionally, as small errors in *D*_max_ can change *P*(*r*) slightly (Grant *et al.*, 2015 ▸), the persistence of features in the face of varying *D*_max_ serves as an additional indicator of the robustness of the solution. Upon further consideration, it is likely that this peak corresponds to vectors contributed by the C-terminal IDRs, which are anchored ∼270 Å apart and should naturally contribute many pairwise distances at *r* > 270 Å (Supplementary Fig. S5). As expected, this peak is not present in the data for SFPQ1–598 (the variant lacking the C-terminal IDRs), which reaches *r* = 0 roughly where the peak begins (Fig. 2 ▸*f*), supporting this point.

For both SFPQ1–707 and SFPQ1–598 the Guinier- and *P*(*r*)-derived *R*_g_ estimations were in agreement and within error margins of each other (Table 1 ▸). For SFPQ1–707 the lowest *q* point measured was 0.00506 Å^−1^ and for SFPQ1–598 it was 0.0047 Å^−1^. Considering the important limit of *q*_min_ < π/*D*_max_ (Kikhney & Svergun, 2015 ▸) and the *D*_max_ values of 434 and 344 Å from our *P*(*r*) analysis of SFPQ1–707 and SFPQ1–598, respectively, the data are within the appropriate *q*-range to resolve species of this size. In theory, this limit and our data range should allow proteins with a maximum size of 620 and 668 Å to be analysed, which is well beyond the size of the longest conformers that have been used to fit the data in our ensemble modelling (474.65 Å for SFPQ1–707 and 362.98 Å for SFPQ1–598; see next section).

The dimensionless Kratky plots for these experiments indicate a progression from globular to partly rod-like to flexible upon the addition of either the N-terminal or both IDRs (Fig. 2 ▸*g*), corroborating the predictions that both the C- and N-terminal IDRs are long, flexible and disordered.

**Figure 3:**
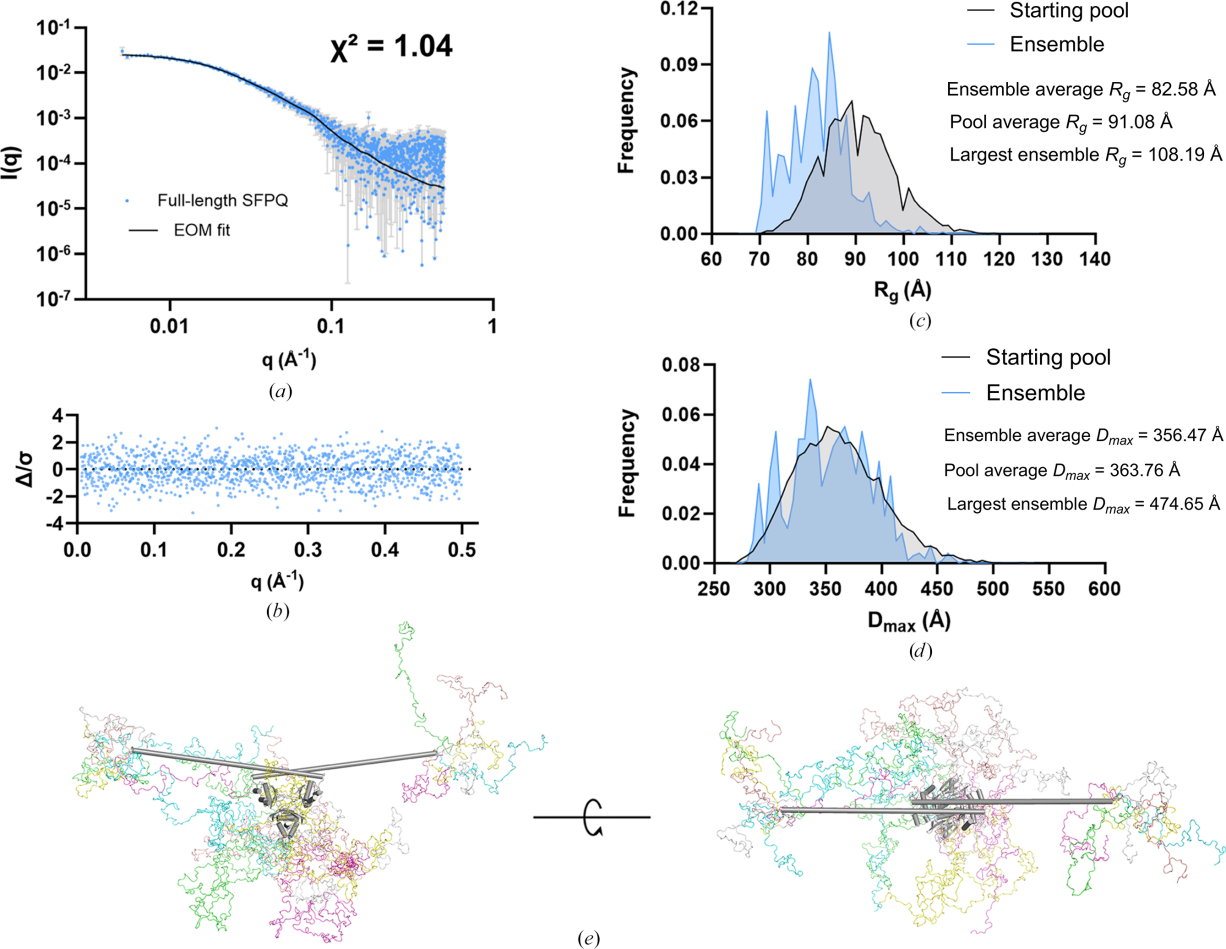

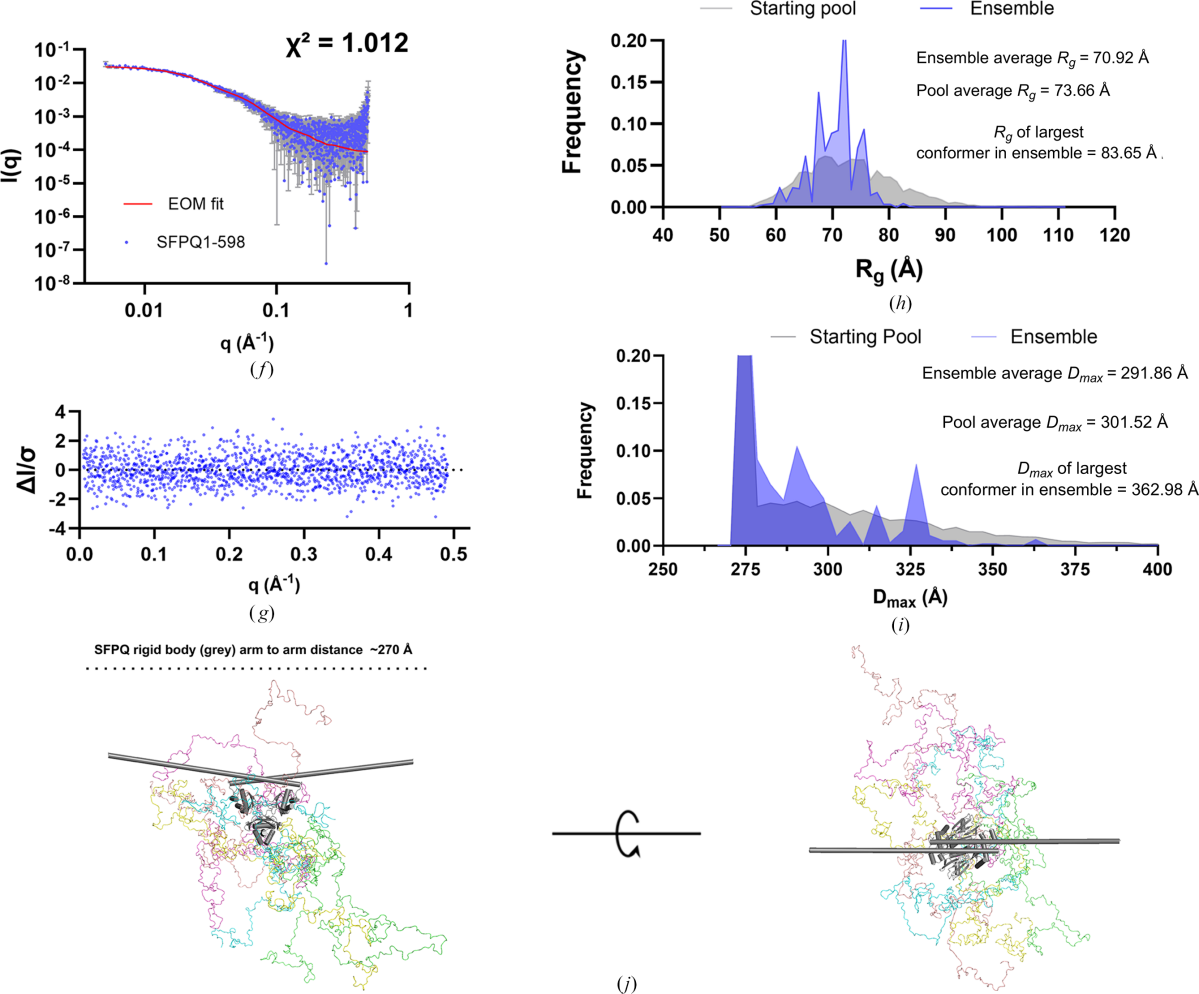
Ensemble modelling of SFPQ using *EOM*: a potential N–C-terminal interaction. (*a*) SEC-SAXS scattering data of full-length SFPQ shown as log(*I*) versus log(*q*). The fit of the *EOM* ensemble is shown as a black line. The χ^2^ of 1.04 indicates an excellent fit to the data. (*b*) Normalized residual plot of the *EOM* fit to experimental data: the lack of systematic variation is indicative of a good fit. (*c*) Frequency versus *R*_g_ plot of the initial random starting pool and the ensembles that fit the data. (*d*) Frequency versus *D*_max_ plot of the initial random starting pool and selected ensembles that fit the data. (*e*) Atomistic models of full-length SFPQ which are from the ensemble that fit the data. (*f*) SEC-SAXS scattering data of SFPQ1–598 as a log(*I*) versus log(*q*) plot. The fit of the *EOM* ensemble is shown as a red line. A χ^2^ of 1.012 indicates an excellent fit to the data. (*g*) Normalized residual plot of the *EOM* fit to the experimental data: the lack of systematic variation is indicative of a good fit. (*h*) Frequency versus *R*_g_ plot of the initial random pool and selected ensembles which fit the data. (*i*) Frequency versus *D*_max_ plot of initial random pools and selected ensembles for SFPQ1–598. (*j*) Selection of models from the ensemble that fit the SFPQ1–598 data.

Given the predicted disordered regions in these proteins and their dimensionless Kratky plots in this study, we used the ensemble-modelling program *EOM* (Tria *et al.*, 2015 ▸), which creates an initial pool of realistically flexible models using a subprogram called *RanCh* (*Random Chains*). The scattering profiles of these models are calculated using *FFMAKER* and a genetic algorithm (*GAJOE*) searches the initial random pool of models for ensembles which together fit the data. The statistics of the best ensembles (usually 50–100 different ensembles) are then pooled and the distribution of their *R*_g_ and *D*_max_ values are compared with the initial random starting pool of models for insight into compaction, flexibility and conformational state. In our analysis, we have termed the ensembles that fit the data simply as ‘ensemble’ and the random initial pool as ‘starting pool’. The resulting models generated by *EOM* had excellent fits to the data, as indicated by near-ideal reduced χ^2^ values for both SFPQ and SFPQ1–598 of 1.04 and 1.012, respectively (Figs. 3 ▸*a* and 3 ▸*f*). The error-weighted residual plots for both experiments also reflected agreement with the experimental data (Figs. 3 ▸*b* and 3 ▸*g*), with no syste­matic variation observed. For full-length SFPQ the filtered selected ensemble of models that fit the data (‘ensemble’) had an average *R*_g_ that was more compact than that of the initial random ensemble of models (‘starting pool’) that was generated by *EOM*. This can be seen by the *R*_g_ ensemble distribution that fits the data shifting left relative to the *RanCh* pool (Fig. 3 ▸*c*). The geometric average of the *R*_g_ distribution for the selected ensemble was 82.58 Å compared with 91.08 Å for the random starting pool, again indicating compaction.

Comparison of the *D*_max_ distributions for the starting pool and the ensemble indicate that they are very similar (Fig. 3 ▸*d*), possibly because of a high number of degrees of freedom in the model. Full-length SFPQ has four disordered domains, perhaps meaning that larger distances can still be reached whilst the average *R*_g_ can simultaneously become smaller. *R*_flex_ of the system is calculated to be ∼74.29%, compared with that of the random pool which is ∼81.12%, with an *R*_σ_ of 0.83, *i.e.* below unity. These results taken together indicate a degree of compaction in the models that fit the data, compared with the initial random pool of conformers, supporting the notion that full-length SFPQ experiences some compaction in solution (Fig. 3 ▸*e*).

### 3.2. Removal of the C-terminal IDR of SFPQ abolishes the preference for chain compaction

For comparison with the *EOM* data on full-length SFPQ, *EOM* was additionally run on the data for SFPQ1–598. However, for SFPQ1–598 the ensemble that fits the data reproduces much of the middle region of the random starting pool in terms of *R*_g_ (Fig. 3 ▸*h*), with the geometric average *R*_g_ values of the selected ensembles and the starting random pools being 70.92 and 73.66 Å, respectively. The distribution of *D*_max_ values selected to fit the data also appears to reproduce the dimensions of the random pool (Fig. 3 ▸*i*). The highest frequency distance is ∼270 Å, as this is the arm-to-arm distance between the long helices in the structured parts of SFPQ. *R*_flex_ and *R*_σ_ reveal that the selected ensembles are not accessing the full conformational space of the random starting pool [*R*_flex_ = 62.04% (∼83.61%) and *R*_σ_ = 0.51]. This is likely to be because the tail ends of the *RanChR*_g_ distribution do not overlap with the selected pool (Fig. 3 ▸*h*), perhaps because the tail ends of the distribution represent more extreme cases of compaction or extension, which do not occur in solution. However, what is obvious is that much of the distribution of ensemble *R_g_* values appears to overlap with the middle of the starting-pool distribution for SFPQ1–598 but is significantly shifted to the left for full-length SFPQ (Figs. 3 ▸*c* and 3 ▸*h*). This is also reflected in the geometric average *R*_g_ values of the respective pools.

A possible explanation for the compaction of the chosen ensembles compared with the starting random pool in the full-length SFPQ experiment is that, as hypothesized by Marshall *et al.* (2023 ▸), the N- and C-terminal IDRs directly interact. This would explain why SFPQ1–598, a variant which lacks the C-terminal IDR, reproduces elements of the random pool more closely and shows less of a preference for more compact conformations. Inspecting the *EOM* models reveals that some models place the C- and N-terminal IDRs in relatively close proximity to one another or show compaction of the C-terminal IDRs back onto the dimer (Fig. 3 ▸*e*). Given that *EOM* models disordered chains realistically using a C^α^–C^α^ Ramachandran distribution in line with that of disordered proteins as well as the user-supplied amino-acid sequence (Tria *et al.*, 2015 ▸), this suggests that it is physically and sterically possible for the IDRs to interact directly. The notion of a direct interaction between the IDRs is also a possible explanation for an additional SANS measurement of full-length SFPQ in a low-salt buffer (150 m*M* KCl) that produced an interesting *P*(*r*) function that appears to be contracted, with a shoulder, compared with full-length SFPQ in high-salt conditions (see Section 3.4; Fig. 5*g*).

### 3.3. The N-terminal IDR of SFPQ collapses at a physiological salt concentration

**Figure 4:**
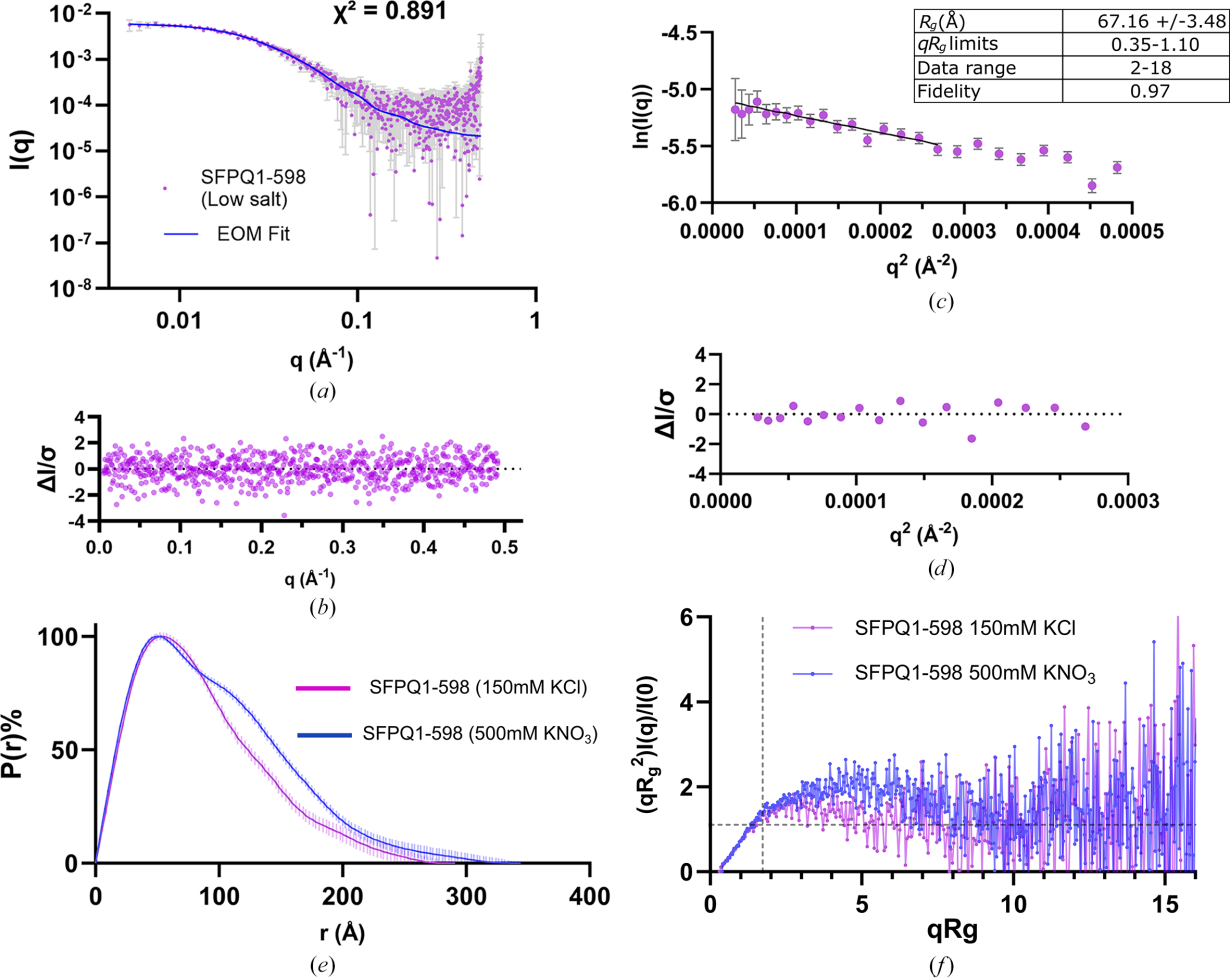

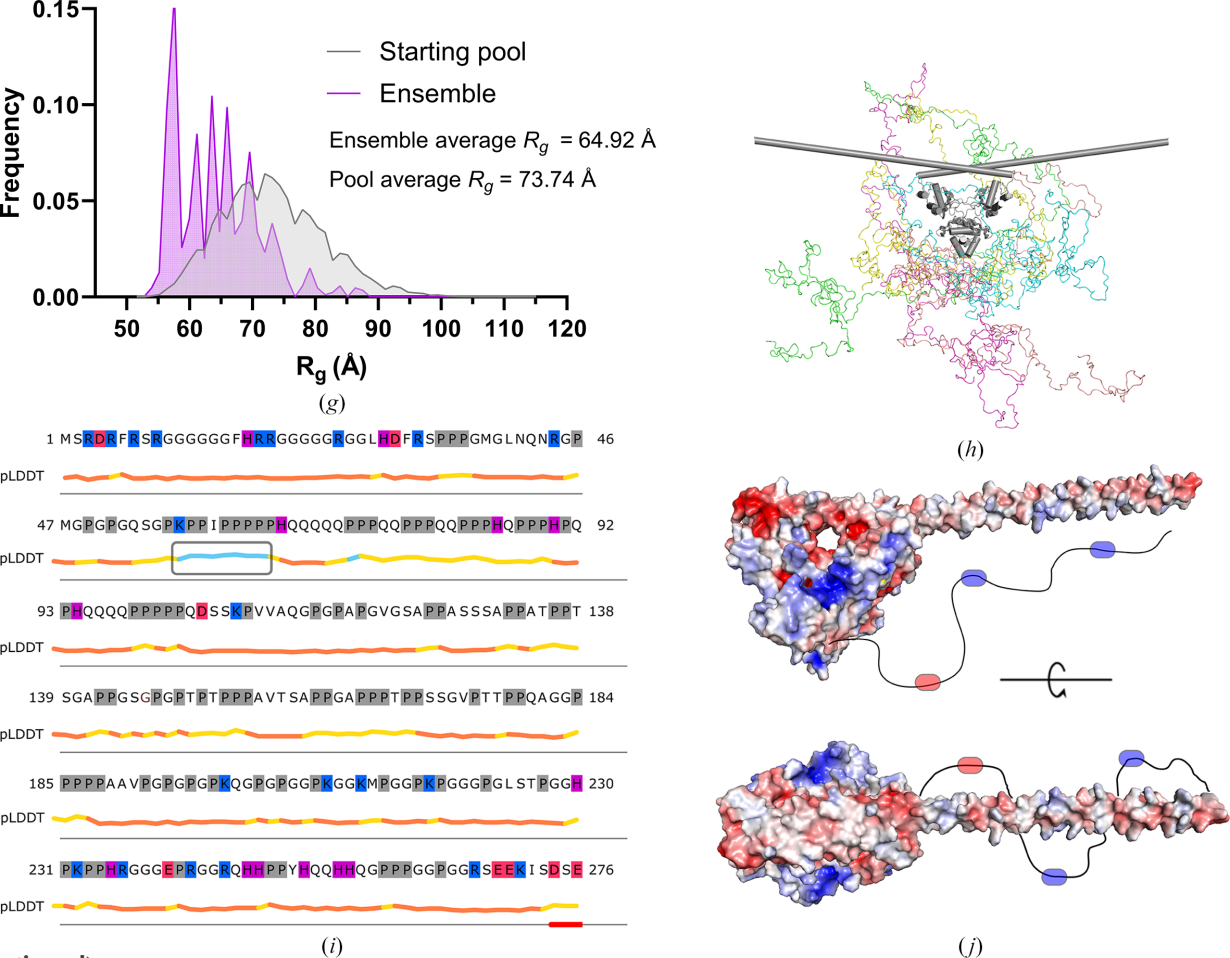
Low-salt versus high-salt data comparison for SFPQ1–598. (*a*) SEC-SAXS scattering data of SFPQ1–598 shown as a log(*I*) versus log(*q*) plot. The fit of the *EOM* ensemble is shown as a blue line. The χ^2^ of 0.891 indicates an excellent fit to the data. (*b*) Normalized residual plot for the *EOM* fit indicating reasonable variation around the fit. (*c*) Guinier analysis indicates a linear fit within the appropriate *qR*_g_ range and an *R*_g_ smaller than that for SFPQ1–598 in high salt. (*d*) The normalized residuals for Guinier analysis indicating reasonable variation of the data around the fit. (*e*) Distance distribution functions of SFPQ1–598 in both salt conditions. (*f*) Dimensionless Kratky analysis comparing SFPQ1–598 in high-salt and low-salt conditions. (*g*) Frequency versus *R*_g_ plot of initial random and selected ensemble pools. (*h*) Atomistic models from the ensemble that fits the data. (*i*) The sequence of the N-terminal IDR of SFPQ (residues 1–276) with charged/proline residues coloured by identity (histidine, purple; arginine and lysine, blue; aspartate and glutamate, red; proline, grey). The *AlphaFold* pLDDT score is shown beneath the sequence, with regions in orange and yellow having a low confidence score and regions in blue having a moderate–high confidence. (*j*) Electrostatic map of an SFPQ homodimer with one of the coiled-coil domains removed for space and simplicity. Blue shading shows positively charged pockets and red shading shows negatively charged pockets. The N-terminal IDR is represented as an unrealistic cartoon line with an alternating charge.

Given the drastic effect of salt concentration observed by Marshall *et al.* (2023 ▸), some experiments on these proteins under different salt conditions were carried out to probe whether charge screening had any measurable effect on the structure of SFPQ. A SEC-SAXS experiment performed on SFPQ1–598 in a lower salt buffer yielded results which differed from the high-salt buffer. SFPQ1–598 is evidently capable of running over a size-exclusion column in a low-salt buffer to some extent, as indicated by the SEC-SAXS data in Fig. 4 ▸(*a*) and Supplementary Fig. S6. *EOM* models indicated good agreement with the experimental data, with a reduced χ^2^ of 0.891 and an error-weighted residuals plot indicating no systematic variability of the fit against the data (Figs. 4 ▸*a* and 4 ▸*b*). These data had a lower Guinier *R*_g_ than that of SFPQ1–598 in the 500 m*M* KNO_3_ buffer (Fig. 4 ▸*c*, Table 1 ▸), with acceptable fit parameters and overall distribution of residuals (Fig. 4 ▸*d*). A comparison of the *P*(*r*) functions between SFPQ1–598 in high- and low-salt conditions reveals a difference in the size of their distributions, whilst maintaining the same approximate shape (Fig. 4 ▸*e*). This difference between salt conditions is also echoed in the dimensionless Kratky plots of both experiments, which reveal that the low-salt condition appears to be more folded compared with the high-salt condition (Fig. 4 ▸*f*). The *EOM* analysis of this condition also supports this, with the *R*_g_ distribution of the selected ensembles shifting significantly to the left compared with that of the random starting pool, with average *R*_g_ values for the selected and random pools of 64.92 and 73.74 Å, respectively. For these data *R*_flex_(random)/*R*_σ_ = ∼70.17 (∼82.68%)/0.80, indicating that the selected ensemble pool does not equally sample the entire conformational space of the random starting pool. Visual inspection of the models also appears to show bunching of the N-terminal IDRs around the dimer core (Fig. 4 ▸*h*). Taken together, the results indicate that changing the buffer from 500 m*M* KNO_3_ to 150 m*M* KCl induces some form of compaction. However, we cannot exclude that the change of counterion could contribute to differences, alongside the change in ion concentration.

Analysis of the sequence of the N-terminal IDR indicates a multitude of basic and acidic amino acids (Fig. 4 ▸*i*). Additionally, an electrostatic potential map of an SFPQ dimer indicates several pockets of positive and negative charge on the surface of the dimerization region as well as the coiled-coil domain (Fig. 4 ▸*j*). Given that the protein noticeably becomes more compact, likely due to reduced charge screening, it may be that these pockets of charge on the surface of the DBHS domain or the alternating regions of charge on the N-terminal IDR allows the collapse of the IDR into a more compact state. This could occur through self-interaction either with the folded DBHS domain or inter-residue contacts within the N-terminal IDR.

### 3.4. Small-angle neutron scattering demonstrates that full-length SFPQ can exchange dimeric partners *in vitro*

**Figure 5:**
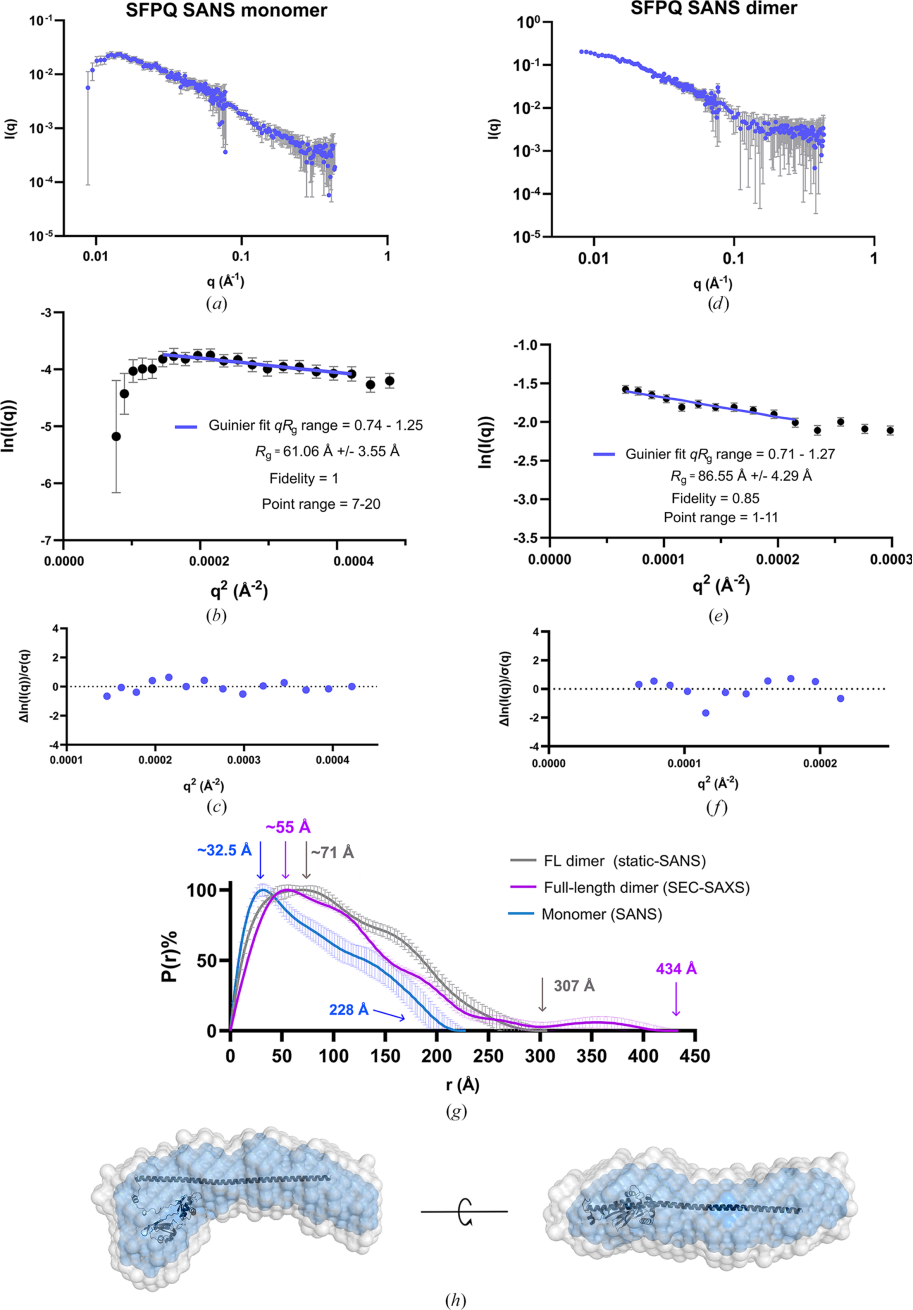
SANS experiments indicating dimer partner exchange between SFPQ homodimers. (*a*) Log(*I*) versus log(*q*) plot for an experiment featuring ∼5% protiated SFPQ (hSFPQ) and 95% deuterated SFPQ (dSFPQ) at a D_2_O match-point of 95%. (*b*) Guinier plot for (*a*) indicating the *qR*_g_ range of 0.74–1.25 with a Guinier *R*_g_ of 61.06 ± 3.55 Å. (*c*) Residual plot of the Guinier fit. (*d*) Log(*I*) versus log(*q*) plot for an experiment featuring ∼5% hSFPQ and 95% dSFPQ in H_2_O without any match-out. (*e*) Guinier plot for (*d*) indicating the *qR*_g_ range of 0.71–1.27 with a Guinier *R*_g_ of 86.55 ± 4.29 Å. (*f*) Residual plot of the Guinier fit from (*e*). (*g*) A comparative *P*(*r*) function plot between full-length SFPQ as observed with SEC-SAXS and the SANS data from these experiments. Differing peak maxima, function shapes and *D*_max_ values indicate that the blue curve corresponds to a monomer of full-length SFPQ. The differing maxima, *D*_max_ values and overall changes in shape between the purple and grey functions may be evidence of the compaction of full-length SFPQ in different salt conditions. (*h*) *DAMAVER* (grey) and *DAMFILT* (blue) envelopes processed from the matched-out SANS data, with an atomistic model of a monomer of SFPQ including just the folded domain superposed over the envelope. This further confirms that the blue function in (*g*) corresponds to a monomer of SFPQ.

Contrast-matching SANS experiments were performed in an attempt to measure the shape of SFPQ in the condensed phase. In our match-out experiment, a significant amount of deuterated protein (95% by ratio) was mixed with a small amount of protiated protein (5% by ratio). This experiment was carried out at the match-point of the deuterated protein (95% D_2_O) such that buffer subtraction should in theory eliminate any contributions to the data from the deuterated protein. Observing SFPQ as a monomer in this instance would indicate dynamic partner exchange between the deuterated proteins, as at this concentration and much lower (see Fig. 5 ▸) SFPQ typically exists as a dimer. Whilst our experiments attempting to study SFPQ inside droplets ultimately failed to produce monodisperse scattering, the assumption that full-length protiated SFPQ and deuterated SFPQ could exchange partners with each other was shown to be correct via a match-out experiment (Figs. 5 ▸*a*, 5 ▸*g* and 5 ▸*h*).

In addition, a data set was collected on a mixture of deuterated and protiated dimers of SFPQ without any match-out conditions, yielding a *P*(*r*) function that could be directly compared with that of full-length SFPQ collected via SEC-SAXS (Figs. 5 ▸*d* and 5 ▸*g*). For the monomer experiment, the data indicated a linear Guinier fit that passed through the error bars of all chosen data points with an *R*_g_ of 61.06 ± 3.55 Å (Fig. 5 ▸*b*) and had an acceptable amount of variation around the fit (Fig. 5 ▸*c*). For the dimer experiment the data also indicated a linear Guinier fit passing through all of the error bars, except for the presence of one point which seemed to deviate from linearity and was likely to be an experimental outlier (Figs. 5 ▸*e* and 5 ▸*f*). This data produced a Guinier *R*_g_ of 86.55 ± 4.29 Å (Fig. 5 ▸*e*), which more or less agrees with the Guinier-derived *R*_g_ of full-length SFPQ from SEC-SAXS (Table 1 ▸ and Fig. 2 ▸). The Guinier fit of these data had an acceptable amount of variation around the fit (Fig. 5 ▸*f*).

However, a comparison of our SAXS and SANS data sets on full-length SFPQ revealed the *P*(*r*) functions from SANS to be shorter than that of the full-length protein as observed with SAXS and to also represent different asymmetric shapes (Fig. 5 ▸*g*). The monomer scattering data set produced a *P*(*r*) function much smaller than both other data sets, as shown by the smallest maxima in *P*(*r*) at ∼32.5 Å and a *D*_max_ of 228 Å (Fig. 5 ▸*g*), which is close to half of the *D*_max_ of SFPQ as seen with SAXS (Fig. 5 ▸*g*). Additionally, an atomistic model of an SFPQ monomer missing the IDRs conformed reasonably well to the shape of the *DAMAVER* envelope derived from the monomer scattering data (Fig. 5 ▸*h*). These data indicate that protiated and deuterated full-length SFPQ are capable of swapping partners dynamically in solution to reach a population of protiated monomers of SFPQ as the predominant scatterer in solution, surrounded by an excess of matched-out deuterated SFPQ. To determine whether deuterated SFPQ was appropriately matched-out in the context of these experiments, measurements were taken of dSFPQ at its match-out point of 95% D_2_O, which yielded scattering consistent with that of the background (Supplementary Fig. S7). Interestingly, a comparison between full-length SFPQ dimers as observed with SEC-SAXS or SANS yields different *P*(*r*) functions. The function for SFPQ from SEC-SAXS has a maximum at ∼55 Å and a *D*_max_ of 434 Å, whereas the function from SANS has a maximum at ∼71 Å and a *D*_max_ of 307 Å.

Given that both functions are of a reasonable quality (Table 1 ▸) and we have demonstrated that the peak in the function for full-length SFPQ between 300 and 434 Å is likely to be a real structural feature, this could be another case of compaction of the protein due to differing experimental conditions. The reduction in *D*_max_ in the low-salt SANS condition and the broadening of the main peak compared with the function derived from SEC-SAXS in high salt is likely to represent contraction or interaction of the IDRs due to reduced electrostatic screening.

### 3.5. Lysine cross-linking mass spectrometry (XL-MS) shows that the N- and C-terminal IDRs both contact the core DBHS region

**Figure 6:**
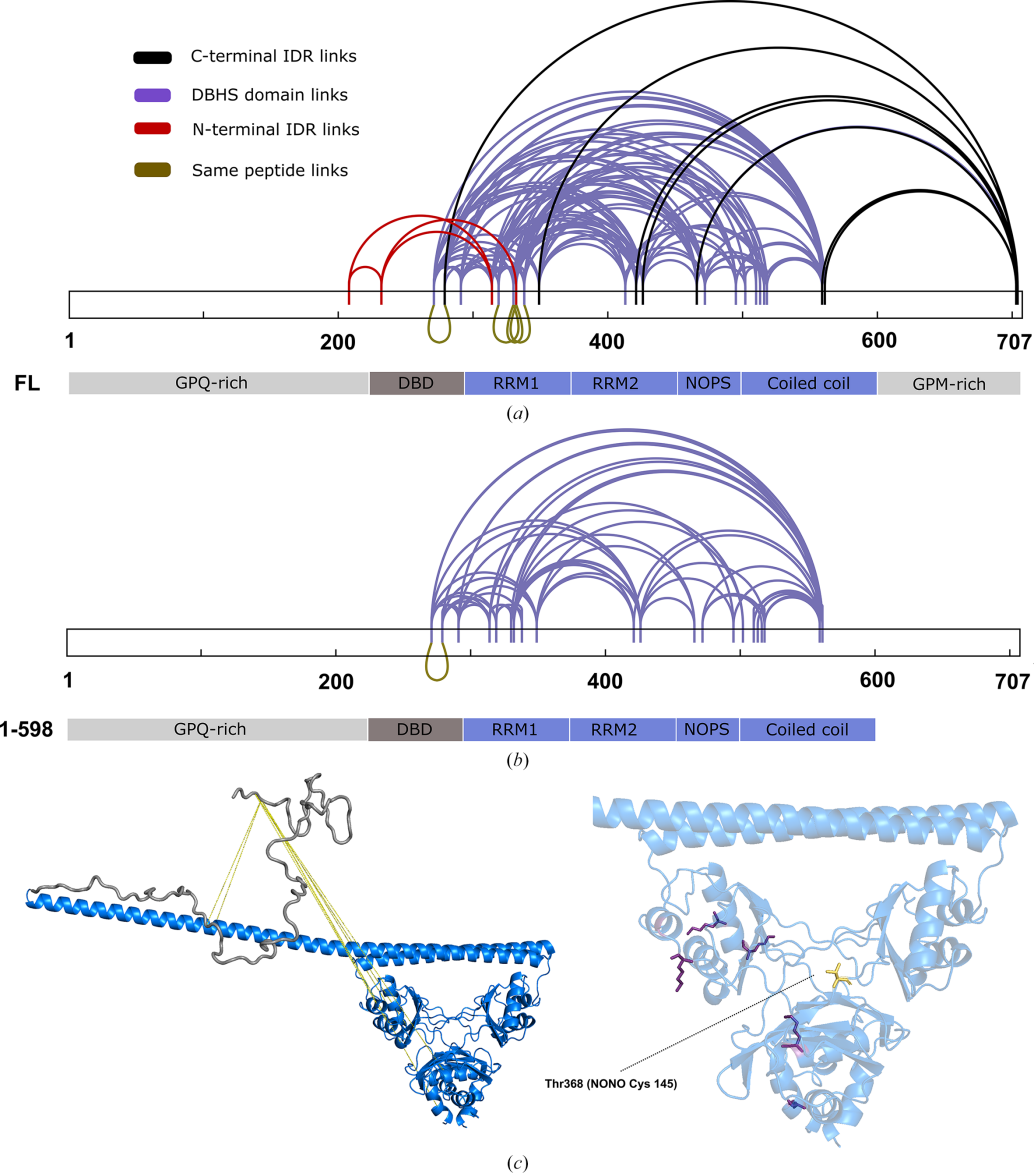
Lysine cross-linking indicates that the C-terminal and N-terminal IDRs make contact with the DBHS domain. (*a*) Lysine cross-links detected via mass spectrometry in full-length SFPQ at 0.7 mg ml^−1^ (10 µ*M*). Cross-links are connected via a line across the amino-acid sequence. Black indicates links involving the C-terminal IDR, purple indicates cross-links within the DBHS domain, red indicates cross-links involving the N-terminal IDR and gold indicates cross-links between the same peptide. (*b*) Cross-links detected for SFPQ1–598 (20 µ*M*). (*c*) The DBHS domain is coloured marine and the C-terminal IDR is coloured grey; points of contact are indicated by a yellow line between the DBHS domain, the coiled-coil domain and the C-terminal IDR. The enlarged DBHS dimer indicates lysines involved in cross-linking (purple). The equivalent position of NONO C145 (Thr368 in SFPQ) has been highlighted in yellow. This may form disulfides with disease-associated cysteine mutants in the C-terminal IDRs of DBHS proteins (see Section 4.4).

In order to obtain information on the intramolecular interactions in play in the context of a dimer of SFPQ, lysine cross-linking mass-spectrometry experiments were performed using full-length SFPQ and SFPQ1–598. The results showed a large number of cross-links forming between the different parts of both protein variants (Figs. 6 ▸*a* and 6 ▸*b*). The significant number of cross-links between regions 276–598 in both data sets is consistent with the large number of lysines close to each other in the structured DBHS region of an SFPQ homodimer (Lee *et al.*, 2015 ▸; Figs. 6 ▸*a* and 6 ▸*b*). To minimize the possibility of self-interaction of SFPQ via the coiled-coil domain (Koning *et al.*, 2025 ▸; Lee *et al.*, 2015 ▸), the experiments were performed at a low concentration (a 10 and 20 µ*M* experiment for each protein variant), which could still provide an interpretable signal for XL-MS. Our additional static SANS measurement supports the notion that at around this concentration dimers are likely to be the only species in solution (Fig. 5 ▸*g*). The only difference is that the initial dilution step for SFPQ in XL-MS was performed in a 500 m*M* KCl buffer, which we would expect to further inhibit self-interaction of the protein, rather than the 150 m*M* KCl buffer for SANS.

Outside of the DBHS domain, cross-links were made between the distal lysines in the C-terminal IDR, the coiled-coil domain and parts of the folded domain, even a partially buried lysine in the dimerization domain (Figs. 6 ▸*a* and 6 ▸*c*). Additional cross-links were also made between the N-terminal IDR and the DBHS domain (Fig. 6 ▸*a*). Both the C-terminal and N-terminal IDRs appear to contact positions on the folded domain in close proximity to one another (Fig. 6 ▸*a*). Despite cross-links not being detected between the two IDRs directly, this demonstrates that there is some overlap in the conformational space that both IDRs can access. Unexpectedly, the N-terminal IDR cross-links with the folded domain disappear in the SFPQ1–598 experiment (Fig. 6 ▸*b*). This could be due to the disruption of an interaction between the C- and N-terminal IDRs which in the full-length protein causes the N-terminal IDR to sample more conformational space near the DBHS domain. A caveat of this experiment is that parts of the N-terminal IDR of SFPQ are highly enriched in proline, which may have resulted in the poor tryptic digest of the region at the distal end of the N-terminus, which did not form any cross-links in either experiment. In theory, this could have led to the subsequent lack of detection of some peptides involving the N-terminal IDR due to a larger undigested mass.

### 3.6. Human DBHS protein sequence bias, enrichment and depletion analysis

**Figure 7:**
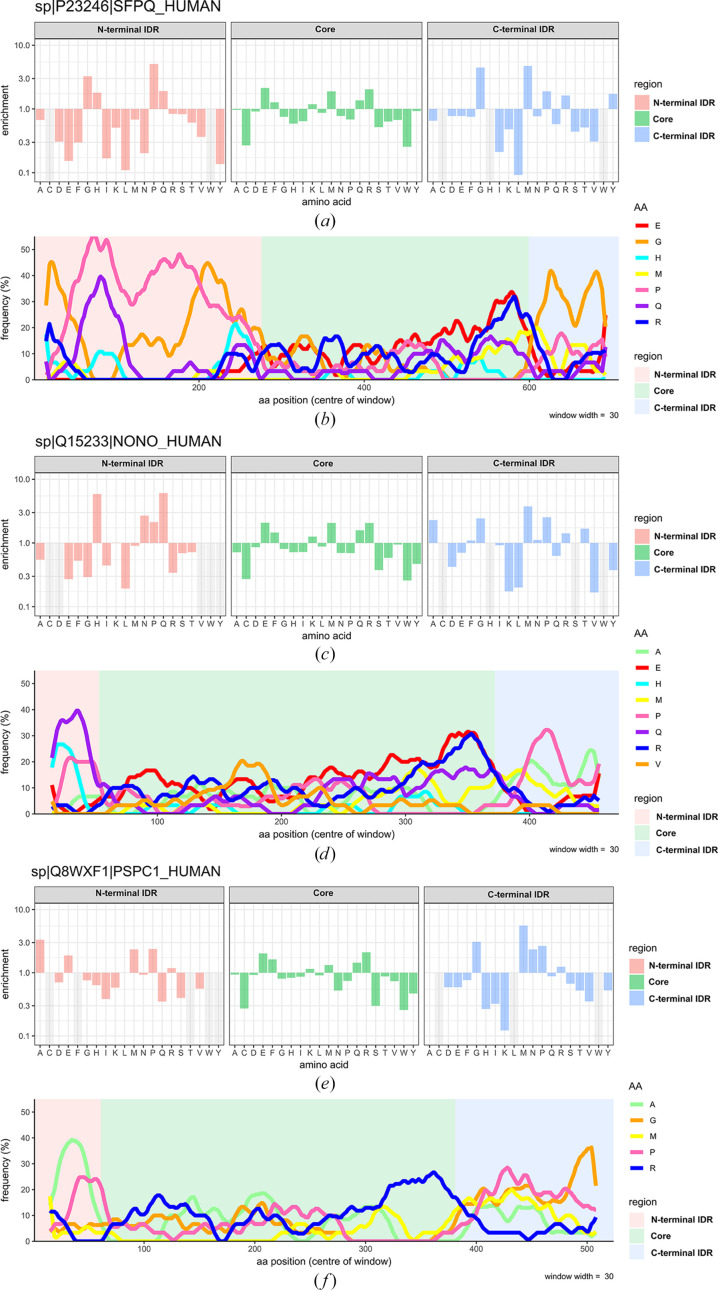
Comparative amino-acid enrichment profiles of the human DBHS paralogs across N-terminal and C-terminal IDRs and the DBHS domain. (*a*) Amino-acid enrichment and depletion histogram of the N- and C-terminal IDRs and the DBHS domain of SFPQ. DBHS sequences are mapped against the average enrichment and depletion of amino acids in the human proteome. (*b*) Amino-acid frequency analysis of the N- and C-terminal IDRs and the DBHS domain of SFPQ using a sliding window of 30 amino acids. (*c*) Amino-acid enrichment and depletion histogram of the N-terminal and C-terminal IDRs and the DBHS domain of NONO mapped against the average enrichment and depletion of the human proteome. (*d*) Amino-acid frequency analysis of the N-terminal and C-terminal IDRs and the DBHS domain of NONO using a sliding window of 30 amino acids. (*e*) Amino-acid enrichment and depletion histogram of the N-terminal and C-terminal IDRs and the DBHS domain of PSPC1 mapped against the average enrichment and depletion of the human proteome. (*f*) Amino-acid frequency analysis of the N-terminal and C-terminal IDRs and the DBHS domain of PSPC1 using a sliding window of 30 amino acids.

To further explore the possible role of combinatorial dimerization and the different DBHS IDRs in the control of LLPS, we analysed the sequence bias and enrichment/depletion of certain amino acids in the different regions of the three human DBHS paralogs (Fig. 7 ▸). The analysis reveals some striking differences in the compositional bias across the IDRs of all of the paralogs. Comparative plots indicate relatively conserved enrichment of amino acids in the DBHS domain across all the paralogs, but highly variable composition across the N- and C-terminal IDRs of the different paralogs (Fig. 7 ▸). Currently, the sequence contribution of each human DBHS paralog to LLPS is poorly understood. These differences and their potential relevance to phase separation and the material properties of different dimeric combinations are discussed in Section 4.

## 4. Discussion

### 4.1. The structure of the N- and C-terminal IDRs and their biological relevance

**Figure 8:**
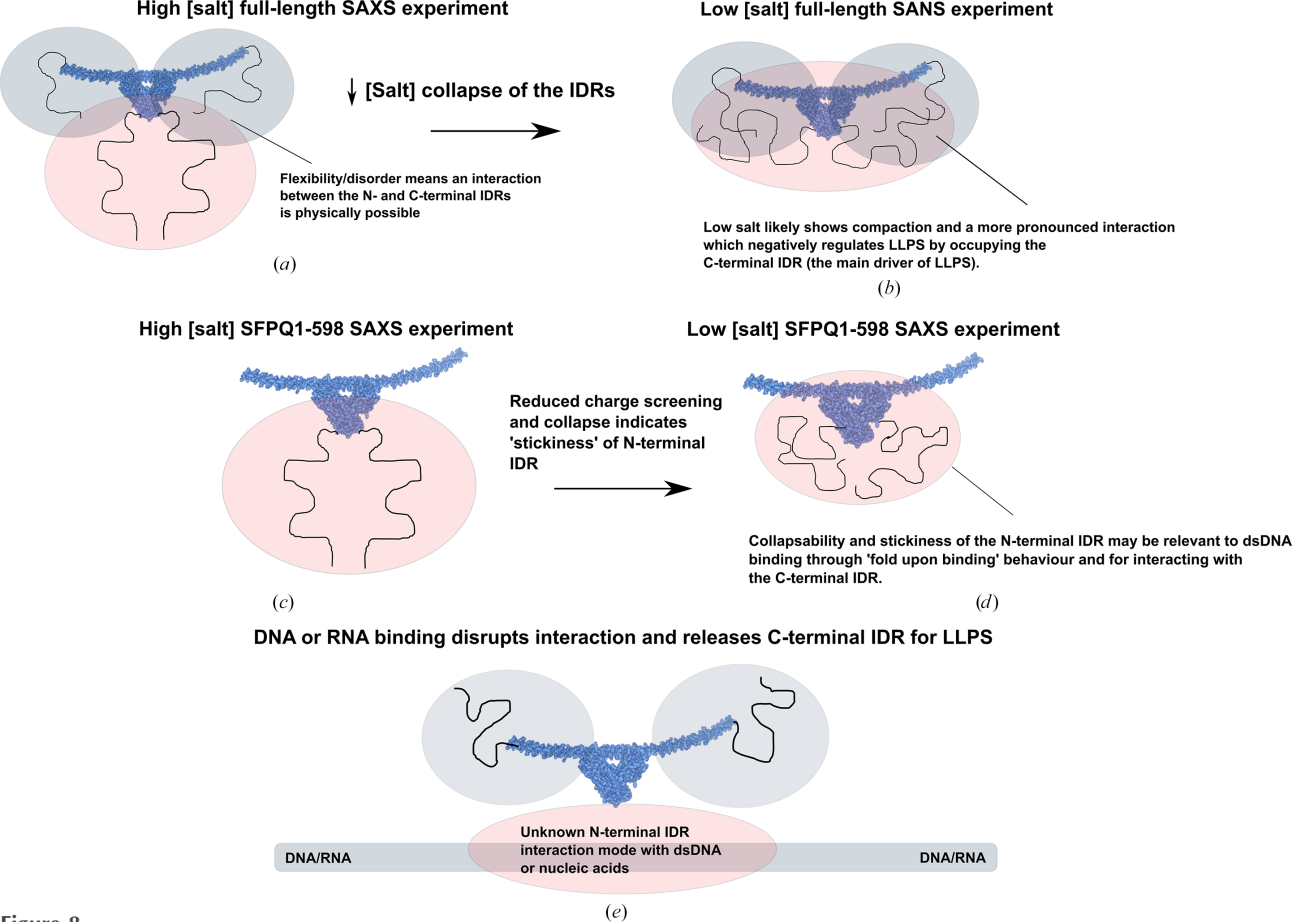
A cartoon model emphasizing the behaviour of SFPQ IDRs based on experimental results. (*a*) SEC-SAXS modelling and XL-MS indicate overlapping conformational space of the N- and C-terminal IDRs, meaning that an interaction between them is possible. (*b*) An additional shorter SANS *P*(*r*) function with a shoulder shows that this interaction is likely to become more pronounced at low salt concentrations. The interaction of the two IDRs is likely to serve to negatively regulate phase separation. The N-terminal IDR can collapse onto itself (*c*, *d*) in response to changing salt concentrations. This ‘stickiness’ may be relevant for the recognition of dsDNA, which may occur in a more structured way where the N-terminal IDR folds upon binding dsDNA or for interactions with the nearby C-terminal IDR. (*e*) The binding of the N-terminal IDR to nucleic acids (long grey bar) would free the C-terminal IDR to drive LLPS. This may act as a trigger that promotes phase separation.

Our data indicate that as per the predictions, the N- and C-terminal regions outside of the DBHS domain of SFPQ are highly flexible in solution and intrinsically disordered. The realistic modelling of the disordered IDRs by *EOM*, combined with the large number of interconverting states that disordered proteins can naturally sample (Holehouse & Kragelund, 2024 ▸), means that it is likely to be physically possible for the IDRs to come into close proximity to one another and interact (Fig. 8 ▸*a*). An interaction between the N- and C-terminal IDRs in SFPQ is a possible explanation for the relative compaction of full-length SFPQ as seen with *EOM*. This interaction, which might be more pronounced at a physiologically relevant salt concentration due to reduced charge screening of the IDRs, might also explain the SANS *P*(*r*) function, which has a larger peak maxima of ∼71 Å, a broader shoulder in the distribution at ∼150–200 Å and a far shorter *D*_max_ of 307 Å compared with the function derived from the SAXS data (Fig. 5 ▸*g*). An interaction between the IDRs is in line with the physical model proposed by Marshall *et al.* (2023 ▸), where the C- and N-terminal IDRs were proposed to interact directly to modulate LLPS (Figs. 8 ▸*a* and 8 ▸*b*). However, it is not necessarily possible to delineate between the compaction of the IDRs or a direct interaction between the two IDRs within the context of this study. Compaction of the C-terminal IDR is also a possibility, given that many points of contact were made between the C-terminal IDR and the DBHS domain in the XL-MS experiments. Perhaps both compaction of the respective IDRs and a direct interaction between them are effects that can occur simultaneously, and these become more exaggerated at a physiological salt concentration due to reduced charge screening. Given the high proline content of the N-terminal IDR (Figs. 7 ▸*a* and 7 ▸*b*), it is also possible that just through the inclusion of the N-terminal IDR on the same structure, and not through direct interaction with the C-terminal IDR, phase separation is hindered due to the well known role of proline as a solubilizing amino acid, which promotes solvation rather than intra-chain interactions (Borcherds *et al.*, 2021 ▸). However, our modelling suggests perhaps otherwise and we have additionally observed the collapse of the N-terminal IDR in low-salt conditions, likely because of intra-chain interactions or interactions with the folded domain.

There have been instances of IDRs which seem extended in high-salt conditions then interacting with pockets of charge on folded RRMs as a result of reduced charge screening (Martin, Thomasen *et al.*, 2021 ▸). It is possible that both the N- and C-terminal IDRs make electrostatic interactions with the pockets of charge on the DBHS domain, creating a complicated balance of direct interactions between the N- and C-terminal IDRs and also the DBHS domain. Given that a symmetry exists between intra-chain interactions in LLPS and inter-chain interactions (Martin, Thomasen *et al.*, 2021 ▸), it is possible that sticker regions (Borcherds *et al.*, 2021 ▸) in the N-terminal IDR cause its collapse and so are also important residues which also may interact with the C-terminal IDR (Figs. 8 ▸*c* and 8 ▸*d*). In this study, we have not attempted to delineate between the interaction of the N-terminal IDR with itself or the folded domain as a cause for its collapse. However, this is worth examining in the future.

Parts of the N-terminal IDR have been shown to be necessary for binding dsDNA (Lee *et al.*, 2015 ▸; Song *et al.*, 2005 ▸; Wang *et al.*, 2022 ▸). This was initially investigated by Urban *et al.* (2002 ▸), who attempted to probe DNA binding through truncations of the N-terminus of SFPQ and concluded that the entire N-terminal IDR could bind DNA. Later studies (Lee *et al.*, 2015 ▸; Wang *et al.*, 2022 ▸) have concentrated the DNA-binding ability of SFPQ to a smaller region within the N-terminal IDR between residues 214 and 298, putatively dubbing it the ‘DNA-binding’ domain. This notion was strengthened by the presence of RGG/RG motifs within the DNA-binding domain (DBD), which are commonly observed in nucleic acid binding (Chong *et al.*, 2018 ▸). However, the distal N-terminal part of the IDR outside the DBD also contains RGG/RG motifs and it is currently unclear whether these are also involved in nucleic acid binding. It is a possibility that RGG tracts outside of the putative DBD in SFPQ are also involved in nucleic acid binding, as in the protein FUS the inclusion of additional disordered RGG motifs to restore mutants of FUS to wild-type FUS enhanced the affinity of the protein for RNA (Ozdilek *et al.*, 2017 ▸). The collapse of the N-terminal IDR that was observed in our modelling is perhaps also relevant to nucleic acid binding. Disordered DNA-binding domains can fold into a more structured conformation when interacting with DNA either via the large-scale folding of entire domains or of more local loops and motifs (Dyson & Wright, 2005 ▸). This may be the case for the interaction of the DBD of SFPQ with certain dsDNA targets (Figs. 8 ▸*d* and 8 ▸*e*) and may be how a low-affinity interaction might stabilize into an interaction with more specificity.

Presuming that the N- and C-terminal IDRs interact directly, it is possible that phase separation might be modulated through further direct interaction of a larger part of the N-terminal IDR (in place of just the DBD) with nucleic acids (Fig. 8 ▸*e*). The binding of a nucleic acid, such as DNA, or larger structured RNA might sequester the N-terminal IDR and leave the C-terminal IDR, the main driver of LLPS (Marshall *et al.*, 2023 ▸) free for interactions with other components (Fig. 8 ▸*e*). Nucleic acids in this sense might act as a further driver or an ‘on-switch’ for phase separation through steric sequestration of the N-terminal IDR, as was also hypothesized by Marshall *et al.* (2023 ▸). This is potentially relevant for the assembly of the initial NEAT1–SFPQ RNP, where SFPQ initially binds core parts of NEAT1 following transcription (West *et al.*, 2016 ▸; Yamazaki *et al.*, 2018 ▸), possibly using both the N-terminal IDR and the RRMs. In theory, this would then free the C-terminal IDR to promote phase separation with other unbound DBHS proteins and seed paraspeckle formation. The competition of nucleic acid targets for SFPQ, which has been demonstrated by Song *et al.* (2005 ▸) and Wang *et al.* (2022 ▸) between VL30 RNA and the GAGE6 oligonucleotide, may also play a role in regulating LLPS. Substituting one target for another might influence the occupancy of the N-terminal IDR and therefore additionally modulate LLPS. Further experimental work is required to assess whether the direct inclusion of nucleic acid targets in LLPS assays can influence the saturation concentration of SFPQ (Marshall *et al.*, 2023 ▸).

### 4.2. Dimer swapping and relevance to phase behaviour

Our study is the first instance in which partner exchange has been demonstrated between full-length homodimers of a DBHS protein, which is remarkable considering the intimate nature of the dimerization core and the extensive set of interactions that make up the dimerization interface (Huang *et al.*, 2018 ▸; Lee *et al.*, 2022 ▸; Passon *et al.*, 2012 ▸) seen in all prior DBHS protein crystal structures. The data shows that full-length SFPQ is capable of swapping partners with itself without the need for cofactors *in vitro*. Structurally, this may occur due to the inherent flexibility/disorder observed in parts of DBHS structures such as the NOPS domain, which could indicate that the coiled-coil domain unravels first and the rest of the structure naturally unfolds and refolds when it finds another partner (Knott *et al.*, 2022 ▸; Lee *et al.*, 2022 ▸). Combinatorial dimerization is likely to serve many purposes such as the differential recognition of nucleic acid targets and protein partners, or as a compensatory mechanism (Lee *et al.*, 2022 ▸; Huang *et al.*, 2018 ▸).

Our amino-acid composition analysis across the human DBHS paralogs indicates a significant difference between the C- and N-terminal IDRs (Fig. 7 ▸). Strikingly, the N-terminal IDR of SFPQ has significant tracts which vary between being 40% and 55% proline (Figs. 7 ▸*a* and 7 ▸*b*). In comparison, the much shorter N-terminal IDRs of NONO and PSPC1 have proline tracts which are closer to ∼25% proline (Figs. 7 ▸*d* and 7 ▸*f*). Interestingly, both the N-terminal IDRs of NONO and PSPC1 are depleted in glycine, which is enriched in SFPQ, which contains glycine-rich tracts (Fig. 7 ▸*b*). Given the roles of proline in chain expansion and solubility (Borcherds *et al.*, 2021 ▸; Lotthammer *et al.*, 2024 ▸), the N-terminal IDR of SFPQ is perhaps more expanded and soluble than the other N-terminal IDRs. This could be further enhanced by its enrichment in glycine, which is known to contribute to IDR flexibility due to the conformational flexibility of the peptide bond (Wang *et al.*, 2018 ▸). Combined with the ∼275 amino-acid length of the N-terminal IDR of SFPQ, the end result is a relatively long, expanded, disordered chain that samples many conformations in space, which is reflected in our modelling data. This is likely relevant to the role of the N-terminal IDR as a nucleic acid-binding domain as well as for interactions with the C-terminal IDR, as increased flexibility and expansion may allow a wider sampling of conformational space, plasticity in the selection of nucleic acid targets and increased contact in solution with the C-terminal IDR to regulate LLPS. Conversely, the lower enrichment of proline, and the depletion of glycine in the N-terminal IDRs of the other paralogs, combined with their significantly shorter length, would contribute to less flexible, compact, IDRs. Intradimer interactions between the N- and C-terminal IDRs may be entirely absent from the other paralogs due to diminished flexibility, shorter domain length and the presence of hydrophobic residues such as alanine (Fig. 7 ▸*f*), which may instead promote self-interaction (Holehouse & Kragelund, 2024 ▸) or work to hinder phase separation. Another striking difference is the enrichment of histidine in the N-terminal IDRs of NONO and SFPQ, which is depleted in the N-terminal IDR of PSPC1 (Fig. 7 ▸). Histidine is capable of π–π stacking and cation–π interactions; in the right context (Liao *et al.*, 2013 ▸) this may be important for interaction with tyrosines, which are enriched in the C-terminal IDR of SFPQ. Recently, King *et al.* (2024 ▸) identified pH-gradient differences across nuclear condensates, with the nucleolus reportedly containing regions of pH 6.5. It is possible that such an effect is more drastic in paraspeckles or other DBHS condensates, and so histidines may contribute to pH sensing in the DBHS IDRs because of their variable protonation state in response to pH.

### 4.3. Compositional differences in the C-terminal IDR;relevance to LLPS

**Figure 9:**
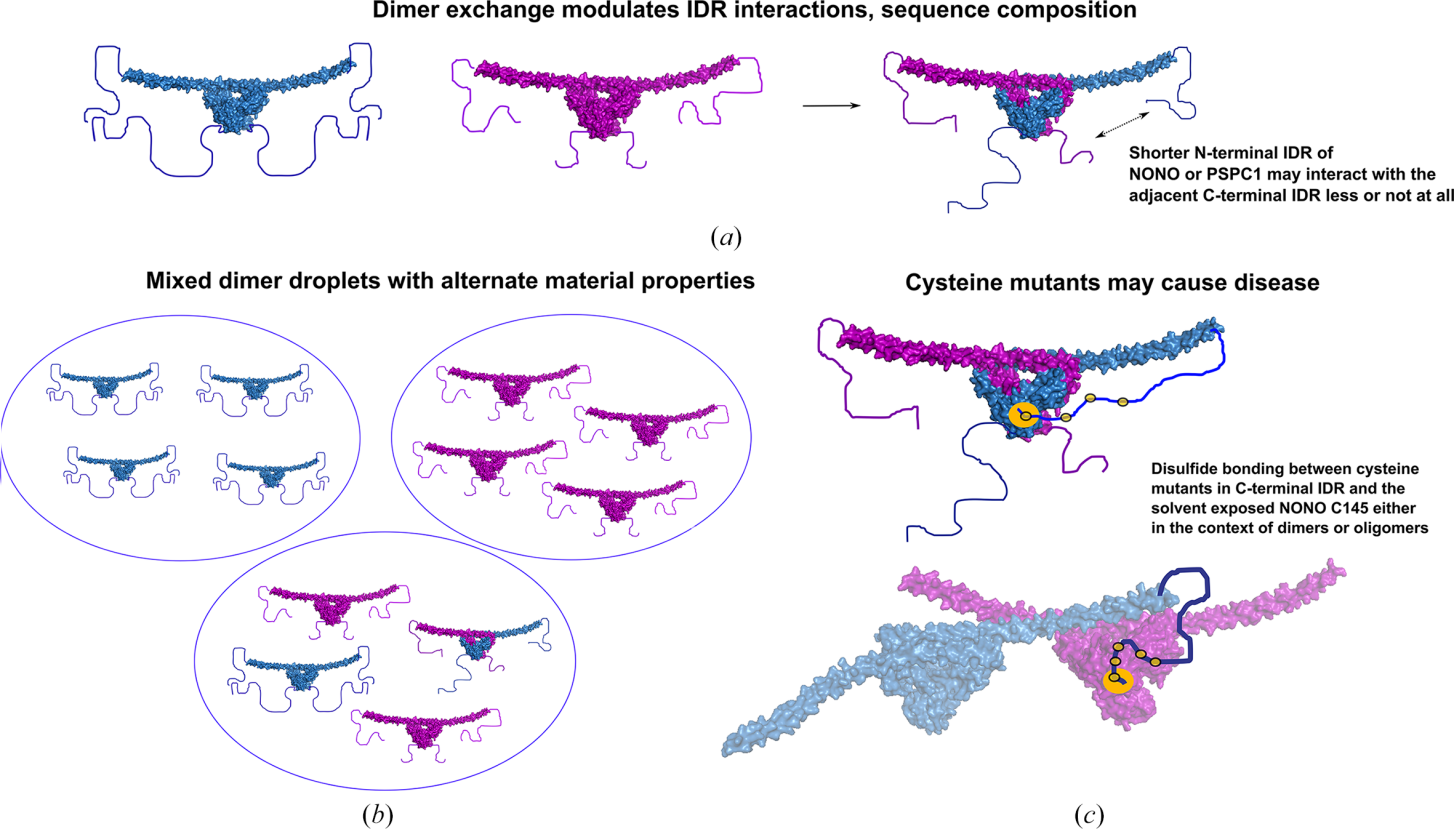
A cartoon summarizing the modulation of phase behaviour through dimer choice and possible mechanisms for disease-associated mutants in the C-terminal IDRs of DBHS proteins. (*a*) Self-interaction of the IDRs of SFPQ as a means to prevent unintended exaggerated phase separation and the possibility for dimer exchange disrupting interactions between IDRs or forming different ones and modulating LLPS. (*b*) Droplets made up of different types of dimers with potentially different material properties. (*c*) Possible mechanism for disease-associated cysteine mutants identified in the C-terminal IDRs of human DBHS proteins. Disulfide bonds could also form directly between IDRs with cysteines in them.

Comparing the C-terminal IDRs of the paralogs also reveals some striking differences, which are of interest given that the C-terminal IDR may be the driver of phase separation for all of the paralogs (Marshall *et al.*, 2023 ▸). SFPQ is enriched in tyrosine (Fig. 7 ▸*a*), which is known to act as a sticker via π–π and cation–π interactions (Bremer *et al.*, 2022 ▸). This, in theory, could contribute to a more compact C-terminal IDR via π–π and cation–π mediated collapse of the chain (Holehouse & Kragelund, 2024 ▸) or an interaction with the histidines or arginines in the N-terminal IDR. Strikingly, tyrosine is depleted in the other paralogs, but phenylalanine, which is also capable of π–π and cation–π interactions (Bremer *et al.*, 2022 ▸), is slightly enriched only in NONO (Fig. 7 ▸*d*). Glycine and proline are enriched in all of the C-terminal IDRs, suggesting chain expansion and flexibility (Lotthammer *et al.*, 2024 ▸). Rather strikingly, alanine tracts feature in both NONO and PSPC1 (Figs. 7 ▸*d* and 7 ▸*f*), but are entirely absent from SFPQ, in which the amino acid is depleted. The departure from tyrosine enrichment in SFPQ to alanine enrichment in NONO and PSPC1 may indicate some reliance on hydrophobic inter­actions for LLPS in NONO and PSPC1 and on π–π and cation–π interactions in SFPQ. Alternatively, alanine tracts may act to hamper phase separation due to their relatively chemically inert nature. An additional interesting point is the conservation and significant enrichment of methionine tracts in the C-terminal IDRs of all of the DBHS proteins (Fig. 7 ▸). Methionine has documented roles in LLPS via its conversion to methionine sulfoxide in response to reactive oxygen species (Aledo, 2021 ▸; Kato *et al.*, 2019 ▸). Given the documented roles of paraspeckles in stress response (McCluggage & Fox, 2021 ▸), methionine sulfoxidation in the DBHS protein IDRs might present a chemical mechanism by which paraspeckles can respond to oxidative stress via post-translational oxidative changes to solvent-exposed methionine tracts. This is particularly interesting given that the methionine enrichment is localized to all of the DBHS C-terminal IDRs, which in SFPQ is the region considered to be the main driver of phase separation. Methionine sulfoxidation would likely alter the chemical properties of the IDR, and as a response alter the phase-separating abilities of all of the DBHS proteins, either promoting or hindering paraspeckle formation. The contributions of the DBHS IDRs to phase separation are complicated and involve many interrelated principles and effects. However, it is likely that dynamic homodimerization and heterodimerization with partner swapping serves to contribute different IDRs to the mixture of interactions that can trigger phase separation and so modulate the material properties of condensates or their occurrence *in vivo* (Figs. 9 ▸*a* and 9 ▸*b*). Further experiments are required to decode the relationship between the sequence composition of IDRs, folded domain behaviour and phase separation.

### 4.4. Disease-associated cysteine mutants in the C-terminal IDRs

As examined in our previous study (Koning *et al.*, 2025 ▸) cysteine mutations in the coiled-coil domain of SFPQ have been shown to cause disulfide oligomerization of the protein. We deduced that due to their structure and flexibility, it might be possible for a variety of cysteine mutations in the C-terminal IDRs of DBHS proteins to also cause disulfide-bound aggregates, which could, in theory, contribute to disease (Fig. 9 ▸*c*).

We have identified numerous cysteine mutants in the C-terminal IDRs of SFPQ, NONO and PSPC1, which we propose may contribute to disease (Supplementary Table S1). Our XL-MS experiments indicate that the C-terminal IDR of SFPQ makes points of contact with the folded DBHS domain, the lysines in which are very close to the solvent-exposed reactive cysteine in NONO C145 (Kathman *et al.*, 2023 ▸). Given the approximate length conservation of the C-terminal IDR between SFPQ and NONO and the longer IDR of PSPC1, it is likely to be possible that all of the paralog IDRs are capable of contact with the DBHS domain. Combined with a capacity for dimer exchange, an SFPQ cysteine IDR mutant might contact the solvent-exposed cysteine in NONO, for example (see the cartoon in Fig. 9 ▸*c*). A cysteine mutation near the middle of the C-terminal IDR of NONO (Reinstein *et al.*, 2016 ▸) has a reported causative role in intellectual disability, presumably through disulfide-bridge formation (Fig. 9 ▸*c*). These ideas may be relevant for other disease states associated with cysteine mutants in the C-terminal IDRs of human DBHS proteins, given the involvement of NONO in certain cancers (Feng *et al.*, 2020 ▸) and the role of SFPQ as a tumour suppressor (Song *et al.*, 2005 ▸).

## 5. Conclusion

Our novel solution scattering studies demonstrate experimentally that the N- and C-terminal IDRs of SFPQ are long, disordered and flexible in solution in accordance with structural predictions. The realistic modelling of disordered chains using *EOM* 2.0 to fit the scattering data suggests that it is physically possible for the IDRs to come close enough to each other to interact in a regulatory manner, as hypothesized by Marshall *et al.* (2023 ▸), which perhaps also explains some of the other features of our data. Such an interaction may have relevance to nucleic acid binding and the formation of condensates, as nucleic acids may work to occupy the N-terminal IDR and disrupt its potential attenuating effect on the C-terminal IDR, thus promoting LLPS. We further demonstrate that full-length protiated SFPQ is capable of swapping dimer partners in solution with other molecules of deuterated SFPQ *in vitro* and that it is possible to capture scattering data of the full-length protein as a monomer using contrast-matching small-angle neutron scattering (SANS). This is the first experimental structural description of the IDRs of SFPQ and their potential dynamics in solution, as well as the capability of full-length SFPQ dimers to exchange partners with each other in a stable manner *in vitro*. These findings are biologically relevant as the IDRs directly control the material state of SFPQ and are either directly or indirectly involved in all of the biological functions of the protein. Additionally, partner swapping between full-length DBHS proteins is likely to allow neofunctionalization of the different subsets of dimers and also the direct modulation of phase properties via the combinations of the different dimers within condensates and the variable IDRs that they contribute to phase separation.

## Supplementary Material

SASBDB reference: full-length SFPQ, SASDV57

SASBDB reference: SFPQ1–598, high salt, SASDV67

SASBDB reference: low salt, SASDV77

SASBDB reference: SFPQ1–707, SANS, monomer, SASDV59

SASBDB reference: dimer, SASDXD4

Supplementary Tables and Figure. DOI: 10.1107/S2059798325005303/ag5054sup1.pdf

## Figures and Tables

**Table 1:** Small-angle X-ray scattering data-collection parameters

	SFPQ1–707 (500 m*M* KNO_3_)	SFPQ1–598 (500 m*M* KNO_3_)	SFPQ1–598 (150 m*M* KCl)	SFPQ1–707, SANS, monomer	SFPQ1–707, SANS, dimer
(*a*) Sample details					
Organism	*Homo sapiens*	*Homo sapiens*	*Homo sapiens*	*Homo sapiens*	*Homo sapiens*
Scattering particle composition	Full-length SFPQ	SFPQ residues 1–598	SFPQ residues 1–598	Full-length SFPQ, 5% protiated and 95% deuterated	Full-length SFPQ, 5% protiated and 95% deuterated
Stoichiometry of components	Single component	Single component	Single component	5:95	5:95
Solvent composition	500 m*M* KNO_3_, 20 m*M* HEPES pH 7.4, 5% glycerol, 1 m*M* DTT	500 m*M* KNO_3_, 20 m*M* HEPES pH 7.4, 5% glycerol, 1 m*M* DTT	150 m*M* KCl, 20 m*M* HEPES pH 7.4, 5% glycerol, 5 m*M* MgCl_2_, 1 m*M* DTT	150 m*M* KCl, 0.075% glycerol, 20 m*M* HEPES, 0.5 m*M* DTT pH 7.4 in 95% D_2_O	150 m*M* KCl, 1.5% glycerol, 20 m*M* HEPES, 1 m*M* DTT pH 7.4 in 100% H_2_O
Sample temperature (°C)	25	25	25	25	25
In-beam sample cell	Co-flow	Co-flow	Co-flow	SANS static cell	SANS static cell
Sample injection concentration (mg ml^−1^)	4.2	5	4.91	2.03 (protiated)	0.63 (deuterated + protiated)
Sample injection volume (ml)	0.06	0.06	0.06	0.325	0.6
SEC column type	Superdex 200 5/150	Superdex 200 5/150	Superdex 200 5/150	Static measurement	Static measurement
SEC flow rate (ml min^−1^)	0.4	0.4	0.4	Static measurement	Static measurement
(*b*) SAS data collection					
Data-acquisition/reduction software	*SCATTERBRAIN* 2.82	*SCATTERBRAIN* 2.82	*SCATTERBRAIN* 2.82	*IGOR Pro*	*IGOR Pro*
Source/instrument description or reference	SAXS/WAXS,Australian Synchrotron	SAXS/WAXS,Australian Synchrotron	SAXS/WAXS,Australian Synchrotron	QUOKKA instrument, ANSTO, Lucas Heights	QUOKKA instrument, ANSTO, Lucas Heights
Wavelength (nm)	0.10781	0.10781	0.10781	0.600	0.600
Camera length (mm)	2790	3000	3000	1300/8000	1300/8000
Measured *q*-range (*q*_min_–*q*_max_; Å^−1^)	0.00506–0.5704	0.0047–0.4900	0.00453–0.4921	0.00815–0.4352	0.00815–0.4352
Method for scaling intensities	Absolute scaling against water	Absolute scaling against water	Absolute scaling against water	Absolute scaling against direct beam	Absolute scaling against direct beam
Exposure time(s), No. of exposures	Frames 132–151 averaged	Frames 147–151 averaged	Frames 148–170 averaged	2 and 3 h	2 and 3 h
(*c*) SAS-derived structural parameters					
Guinier analysis methods/software	*ATSAS* 4.0	*ATSAS* 4.0	*ATSAS* 4.0	*ATSAS* 4.0	*ATSAS* 4.0
Guinier *I*(0) ± σ (cm^−1^)	0.027 ± 0.00052	0.034 ± 0.00095	0.0062 ± 0.00017	0.029 ± 0.0012	0.24 ± 0.0083
Guinier *R*_g_ ± σ (Å)	88.93 ± 2.98	82.44 ± 4.29	67.16 ± 3.48	61.06 ± 3.55	86.55 ± 4.29
Guinier min < *qR*_g_ < max limit (or data-point range)	0.52–1.15	0.42–1.11	0.35–1.10	0.74–1.25	0.71–1.27
Linear fit assessment (fidelity in *PRIMUS*)	0.73	0.56	0.97	1	0.85
Point range	3–22	2–26	2–18	7–20	1–11
PDDF/*P*(*r*) analysis	*ATSAS* 3.2.1	*ATSAS* 3.2.1	*ATSAS* 3.2.1	*ATSAS* 3.2.1	*ATSAS* 3.2.1
*P*(*r*) *I*(0) ± σ (cm^−1^)	0.0271 ± 0.0009	0.03367 ± 0.000786	0.006288 ± 0.0001915	0.02930 ± 0.001992	0.2330 ± 0.07832
*P*(*r*) *R*_g_ ± σ (Å)	93.53 ± 7.82	80.16 ± 4.076	70.52 ± 3.558	66.70 ± 4.678	85.97 ± 3.945
*D*_max_ (Å)	434	344	281	228	307
*P*(*r*) *q*-range/point range (Å^−1^)	0.0051–0.1007 (1–256)	0.0051–0.1089 (1–299)	0.0059–0.1321 (2–183)	0.012–0.1299 (7–132)	0.0081–0.0914 (1–122)
*P*(*r*) fit assessment (total quality estimate)	0.69 (reasonable)	0.71 (reasonable)	0.75 (reasonable)	0.67 (reasonable)	0.78 (good)
α	0.53	1.9	0.93	0.1341	0.27
(*d*) Scattering particle size					
Methods/software	Fischer method	Fischer method	Fischer method	Fischer method	Fischer method
Volume (Å^3^)	251000	217000	202000	117000	241000
Molecular-weight estimate from chemical composition (kDa)	152.554 (dimer)	130.298 (dimer)	130.298 (dimer)	76.27 (monomer)	152.554 (dimer)
Molecular-weight estimate from SAS, concentration-independent method (Fischer method) (kDa)	206	178	166	96	197
(*e*) Data deposition					
SASBDB code	SASDV57	SASDV67	SASDV77	SASDV59	SASDXD4

## References

[bb1] Aledo, J. C. (2021). *Biomolecules*, **11**, 1248. 10.3390/biom11081248 PMC8394241 34439914

[bb2] Borcherds, W., Bremer, A., Borgia, M. B. & Mittag, T. (2021). *Curr. Opin. Struct. Biol.* **67**, 41–50. 10.1016/j.sbi.2020.09.004 PMC8044266 33069007

[bb3] Bremer, A., Farag, M., Borcherds, W. M., Peran, I., Martin, E. W., Pappu, R. V. & Mittag, T. (2022). *Nat. Chem.* **14**, 196–207. 10.1038/s41557-021-00840-w PMC8818026 34931046

[bb4] Chong, P. A., Vernon, R. M. & Forman-Kay, J. D. (2018). *J. Mol. Biol.* **430**, 4650–4665. 10.1016/j.jmb.2018.06.014 29913160

[bb5] Combe, C. W., Graham, M., Kolbowski, L., Fischer, L. & Rappsilber, J. (2024). *J. Mol. Biol.* **436**, 168656. 10.1016/j.jmb.2024.168656 39237202

[bb6] Dayhoff, G. W. II & Uversky, V. N. (2022). *Protein Sci.* **31**, e4496. 10.1002/pro.4496 PMC9679974 36334049

[bb7] Duff, A. P., Wilde, K. L., Rekas, A., Lake, V. & Holden, P. J. (2015). *Methods Enzymol.* **565**, 3–25. 10.1016/bs.mie.2015.06.014 26577725

[bb8] Dyson, H. J. & Wright, P. E. (2005). *Nat. Rev. Mol. Cell Biol.* **6**, 197–208. 10.1038/nrm1589 15738986

[bb9] Feng, P., Li, L., Deng, T., Liu, Y., Ling, N., Qiu, S., Zhang, L., Peng, B., Xiong, W., Cao, L., Zhang, L. & Ye, M. (2020). *J. Cell. Mol. Med.* **24**, 4368–4376. 10.1111/jcmm.15141 PMC7176863 32168434

[bb99] Fischer, H., de Oliveira Neto, M., Napolitano, H. B., Polikarpov, I. & Craievich, A. F. (2010). *J. Appl. Cryst.* **43**, 101–109.

[bb11] Fox, A. H., Nakagawa, S., Hirose, T. & Bond, C. S. (2018). *Trends Biochem. Sci.* **43**, 124–135. 10.1016/j.tibs.2017.12.001 29289458

[bb13] Grant, T. D., Luft, J. R., Carter, L. G., Matsui, T., Weiss, T. M., Martel, A. & Snell, E. H. (2015). *Acta Cryst.* D**71**, 45–56. 10.1107/S1399004714010876 PMC4304685 25615859

[bb14] Hatos, A., Tosatto, S. C. E., Vendruscolo, M. & Fuxreiter, M. (2022). *Nucleic Acids Res.* **50**, W337–W344. 10.1093/nar/gkac386 PMC9252777 35610022

[bb16] Hewage, T. W., Caria, S. & Lee, M. (2019). *Acta Cryst.* F**75**, 439–449. 10.1107/S2053230X19006599 PMC6572092 31204691

[bb17] Holehouse, A. S. & Kragelund, B. B. (2024). *Nat. Rev. Mol. Cell Biol.* **25**, 187–211. 10.1038/s41580-023-00673-0 PMC11459374 37957331

[bb19] Huang, J., Casas Garcia, G. P., Perugini, M. A., Fox, A. H., Bond, C. S. & Lee, M. (2018). *J. Biol. Chem.* **293**, 6593–6602. 10.1074/jbc.RA117.001451 PMC5925804 29530979

[bb20] Kao, A., Chiu, C. L., Vellucci, D., Yang, Y., Patel, V. R., Guan, S., Randall, A., Baldi, P., Rychnovsky, S. D. & Huang, L. (2012). *Mol. Cell. Proteomics*, **10**, M110.002212. 10.1074/mcp.M110.002212 PMC3013449 20736410

[bb21] Kathman, S. G., Koo, S. J., Lindsey, G. L., Her, H. L., Blue, S. M., Li, H., Jaensch, S., Remsberg, J. R., Ahn, K., Yeo, G. W., Ghosh, B. & Cravatt, B. F. (2023). *Nat. Chem. Biol.* **19**, 825–836. 10.1038/s41589-023-01270-0 PMC10337234 36864190

[bb22] Kato, M., Yang, Y. S., Sutter, B. M., Wang, Y., McKnight, S. L. & Tu, B. P. (2019). *Cell*, **177**, 711–721. 10.1016/j.cell.2019.02.044 PMC6752730 30982603

[bb23] Kikhney, A. G., Borges, C. R., Molodenskiy, D. S., Jeffries, C. M. & Svergun, D. I. (2020). *Protein Sci.* **29**, 66–75. 10.1002/pro.3731 PMC6933840 31576635

[bb24] Kikhney, A. G. & Svergun, D. I. (2015). *FEBS Lett.* **589**, 2570–2577. 10.1016/j.febslet.2015.08.027 26320411

[bb25] King, M. R., Ruff, K. M., Lin, A. Z., Pant, A., Farag, M., Lalmansingh, J. M., Wu, T., Fossat, M. J., Ouyang, W., Lew, M. D., Lundberg, E., Vahey, M. D. & Pappu, R. V. (2024). *Cell*, **187**, 1889–1906. 10.1016/j.cell.2024.02.029 PMC11938373 38503281

[bb26] Kirby, N., Cowieson, N., Hawley, A. M., Mudie, S. T., McGillivray, D. J., Kusel, M., Samardzic-Boban, V. & Ryan, T. M. (2016). *Acta Cryst.* D**72**, 1254–1266. 10.1107/S2059798316017174 PMC5137223 27917826

[bb27] Knott, G. J., Bond, C. S. & Fox, A. H. (2016). *Nucleic Acids Res.* **44**, 3989–4004. 10.1093/nar/gkw271 PMC4872119 27084935

[bb28] Knott, G. J., Chong, Y. S., Passon, D. M., Liang, X., Deplazes, E., Conte, M., Marshall, A., Lee, M., Fox, A. & Bond, C. (2022). *Nucleic Acids Res.* **50**, 522–535. 10.1093/nar/gkab1216 PMC8754649 34904671

[bb29] Koenigsberg, A. L. & Heldwein, E. E. (2018). *J. Biol. Chem.* **293**, 15827–15839. 10.1074/jbc.RA118.004481 PMC6187633 30166339

[bb30] Koning, H. J., Lai, J. Y., Marshall, A. C., Stroeher, E., Monahan, G., Pullakhandam, A., Knott, G. J., Ryan, T. M., Fox, A. H., Whitten, A., Lee, M. & Bond, C. S. (2025). *Nucleic Acids Res.* **53**, gkae1198. 10.1093/nar/gkae1198 PMC11754644 39698821

[bb33] Lancaster, A. K., Nutter-Upham, A., Lindquist, S. & King, O. D. (2014). *Bioinformatics*, **30**, 2501. 10.1093/bioinformatics/btu310 PMC4147883 24825614

[bb34] Lee, M., Sadowska, A., Bekere, I., Ho, D., Gully, B. S., Lu, Y., Iyer, K. S., Trewhella, J., Fox, A. H. & Bond, C. S. (2015). *Nucleic Acids Res.* **43**, 3826–3840. 10.1093/nar/gkv156 PMC4402515 25765647

[bb35] Lee, P. W., Marshall, A. C., Knott, G. J., Kobelke, S., Martelotto, L., Cho, E., McMillan, P. J., Lee, M., Bond, C. S. & Fox, A. H. (2022). *J. Biol. Chem.* **298**, 102563. 10.1016/j.jbc.2022.102563 PMC9643411 36209820

[bb37] Liao, S.-M., Du, Q.-S., Meng, J.-Z., Pang, Z.-W. & Huang, R.-B. (2013). *Chem. Cent. J.* **7**, 44. 10.1186/1752-153X-7-44 PMC3599372 23452343

[bb38] Lim, Y. W., James, D., Huang, J. & Lee, M. (2020). *Int. J. Mol. Sci.* **21**, 7151. 10.3390/ijms21197151 PMC7582472 32998269

[bb39] Liu, F., Lössl, P., Scheltema, R., Viner, R. & Heck, A. J. R. (2017). *Nat. Commun.* **8**, 15473. 10.1038/ncomms15473 PMC5454533 28524877

[bb40] Lotthammer, J. M., Ginell, G. M., Griffith, D., Emenecker, R. J. & Holehouse, A. S. (2024). *Nat. Methods*, **21**, 465–476. 10.1038/s41592-023-02159-5 PMC10927563 38297184

[bb41] Manalastas-Cantos, K., Konarev, P. V., Hajizadeh, N. R., Kikhney, A. G., Petoukhov, M. V., Molodenskiy, D. S., Panjkovich, A., Mertens, H. D. T., Gruzinov, A., Borges, C., Jeffries, C. M., Svergun, D. I. & Franke, D. (2021). *J. Appl. Cryst.* **54**, 343–355. 10.1107/S1600576720013412 PMC7941305 33833657

[bb42] Marshall, A. C., Cummins, J., Kobelke, S., Zhu, T., Widagdo, J., Anggono, V., Hyman, A., Fox, A. H., Bond, C. S. & Lee, M. (2023). *J. Mol. Biol.* **435**, 168364. 10.1016/j.jmb.2023.168364 37952770

[bb43] Martin, E. W., Hopkins, J. B. & Mittag, T. (2021). *Methods Enzymol.* **646**, 185–222 10.1016/bs.mie.2020.07.002 PMC8370720 33453925

[bb44] Martin, E. W., Thomasen, F. E., Milkovic, N. M., Cuneo, M. J., Grace, C. R., Nourse, A., Lindorff-Larsen, K. & Mittag, T. (2021). *Nucleic Acids Res.* **49**, 2931–2945. 10.1093/nar/gkab063 PMC7969017 33577679

[bb45] McCluggage, F., Fox, A. H. (2021). *Bioessays*, **43**, e2000245. 10.1002/bies.202000245 33748979

[bb47] Mirdita, M., Schütze, K., Moriwaki, Y., Heo, L., Ovchinnikov, S. & Steinegger, M. (2022). *Nat. Methods*, **19**, 679–682. 10.1038/s41592-022-01488-1 PMC9184281 35637307

[bb49] Ozdilek, B. A., Thompson, V. F., Ahmed, N. S., White, C. I., Batey, R. T. & Schwartz, J. C. (2017). *Nucleic Acids Res.* **45**, 7984–7996. 10.1093/nar/gkx460 PMC5570134 28575444

[bb50] Passon, D. M., Lee, M., Rackham, O., Stanley, W. A., Sadowska, A., Filipovska, A., Fox, A. H. & Bond, C. S. (2012). *Proc. Natl Acad. Sci. USA*, **109**, 4846–4850. 10.1073/pnas.1120792109 PMC3324020 22416126

[bb51] Petoukhov, M. V., Franke, D., Shkumatov, A. V., Tria, G., Kikhney, A. G., Gajda, M., Gorba, C., Mertens, H. D. T., Konarev, P. V. & Svergun, D. I. (2012). *J. Appl. Cryst.* **45**, 342–350. 10.1107/S0021889812007662 PMC4233345 25484842

[bb52] Reinstein, E., Tzur, S., Cohen, R., Bormans, C. & Behar, D. M. (2016). *Eur. J. Hum. Genet.* **24**, 1635–1638. 10.1038/ejhg.2016.72 PMC5110068 27329731

[bb53] Ryan, T. M., Trewhella, J., Murphy, J. M., Keown, J. R., Casey, L., Pearce, F. G., Goldstone, D. C., Chen, K., Luo, Z., Kobe, B., McDevitt, C. A., Watkin, S. A., Hawley, A. M., Mudie, S. T., Samardzic Boban, V. & Kirby, N. (2018). *J. Appl. Cryst.* **51**, 97–111.

[bb54] Schell, B., Legrand, P. & Fribourg, S. (2022). *Biochimie*, **198**, 1–7 10.1016/j.biochi.2022.02.011 35245601

[bb55] Sethi, A., Rawlinson, S. M., Dubey, A., Ang, C. S., Choi, Y. H., Yan, F., Okada, K., Rozario, A. M., Brice, A. M., Ito, N., Williamson, N. A., Hatters, D. M., Bell, T. D. M., Arthanari, H., Moseley, G. W. & Gooley, P. R. (2023). *Proc. Natl Acad. Sci. USA*, **120**, e2217066120. 10.1073/pnas.2217066120 PMC10083601 36989298

[bb56] Song, X., Sun, Y. & Garen, A. (2005). *Proc. Natl Acad. Sci. USA*, **102**, 12189–12193. 10.1073/pnas.0505179102 PMC1189330 16079199

[bb57] Stachowski, T. R., Snell, M. E. & Snell, E. H. (2021). *J. Synchrotron Rad.* **28**, 1309–1320. 10.1107/S1600577521004045 PMC8415334 34475280

[bb58] Svergun, D. I. (1992). *J. Appl. Cryst.* **25**, 495–503.

[bb59] Takeuchi, A., Iida, K., Tsubota, T., Hosokawa, M., Denawa, M., Brown, J. B., Ninomiya, K., Ito, M., Kimura, H., Abe, T., Kiyonari, H., Ohno, K. & Hagiwara, M. (2018). *Cell Rep.* **23**, 1326–1341. 10.1016/j.celrep.2018.03.141 29719248

[bb60] Trewhella, J., Duff, A. P., Durand, D., Gabel, F., Guss, J. M., Hendrickson, W. A., Hura, G. L., Jacques, D. A., Kirby, N. M., Kwan, A. H., Pérez, J., Pollack, L., Ryan, T. M., Sali, A., Schneidman-Duhovny, D., Schwede, T., Svergun, D. I., Sugiyama, M., Tainer, J. A., Vachette, P., Westbrook, J. & Whitten, A. E. (2017). *Acta Cryst.* D**73**, 710–728. 10.1107/S2059798317011597 PMC5586245 28876235

[bb61] Trewhella, J., Jeffries, C. M. & Whitten, A. E. (2023). *Acta Cryst.* D**79**, 122–132. 10.1107/S2059798322012141 PMC9912924 36762858

[bb62] Tria, G., Mertens, H. D. T., Kachala, M. & Svergun, D. I. (2015). *IUCrJ*, **2**, 207–217. 10.1107/S205225251500202X PMC4392415 25866658

[bb64] Urban, R. J., Bodenburg, Y. H. & Wood, T. G. (2002). *Am. J. Physiol. Endocrinol. Metab.* **283**, E423–E427. 10.1152/ajpendo.00057.2002 12169434

[bb65] Vickers, T. A. & Crooke, S. T. (2016). *PLoS One*, **11**, e0161930. 10.1371/journal.pone.0161930 PMC5003356 27571227

[bb66] Wang, J. A., Choi, J., Holehouse, A. S., Lee, H. O., Zhang, X., Jahnel, M., Maharana, S., Lemaitre, R., Pozniakovsky, A., Drechsel, D., Poser, I., Pappu, R. V., Alberti, S. & Hyman, A. A. (2018). *Cell*, **174**, 688–699. 10.1016/j.cell.2018.06.006 PMC6063760 29961577

[bb67] Wang, J., Sachpatzidis, A., Christian, T. D., Lomakin, I. B., Garen, A. & Konigsberg, W. H. (2022). *Biochemistry*, **61**, 1723–1734. 10.1021/acs.biochem.2c00192 35998361

[bb68] West, J. A., Mito, M., Kurosaka, S., Takumi, T., Tanegashima, C., Chujo, T., Yanaka, K., Kingston, R. E., Hirose, T., Bond, C., Fox, A. & Nakagawa, S. (2016). *J. Cell Biol.* **214**, 817–830. 10.1083/jcb.201601071 PMC5037409 27646274

[bb69] Whitten, A. E., Cai, S. & Trewhella, J. (2008). *J. Appl. Cryst.* **41**, 222–226.

[bb70] Yamazaki, T., Souquere, S., Chujo, T., Kobelke, S., Chong, Y. S., Fox, A. H., Bond, C. S., Nakagawa, S., Pierron, G. & Hirose, T. (2018). *Mol. Cell*, **70**, 1038–1053. 10.1016/j.molcel.2018.05.019 29932899

[bb71] Zheng, W. & Best, R. B. (2018). *J. Mol. Biol.* **430**, 2540–2553. 10.1016/j.jmb.2018.03.007 PMC6093220 29571687

