# Supplementary material for: Structural dynamics of IDR interactions in human SFPQ and implications for liquid–liquid phase separation
Source: Acta Crystallogr D Struct Biol. 2025 Jun 27;81(Pt 7):357–79. doi: 10.1107/S2059798325005303 (PMC12216677; doi:10.1107/S2059798325005303)
Supplement: Supplementary file 1 [file d-81-00357-sup1.pdf]

STRUCTURAL BIOLOGY

Volume 81 (2025)

Supporting information for article:

Structural dynamics of IDR interactions in human SFPQ and implications for liquid–liquid phase separation

Heidar J. Koning, Valerie Lai, Ashish Sethi, Shatabdi Chakraborty, Ching-Seng Ang, Archa H. Fox, Anthony P. Duff, Andrew E. Whitten, Andrew C. Marshall and Charles S. Bond

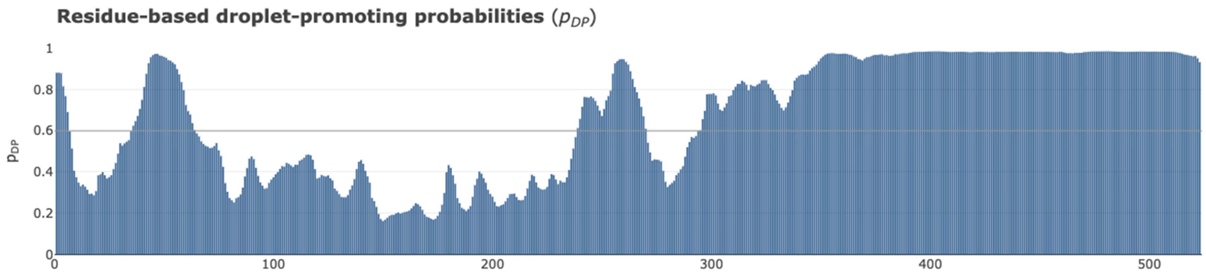

Figure S1 PSPC1 droplet promoting abilities shown as Per residue droplet promoting score (Pdp) as a function of the sequence of PSPC1. Both N and C-terminal IDRs predicted to be capable of promoting phase separation. N-terminal alanine tracts are consistent with a reduction in the predicted ability to phase separate, residues ~1-50.

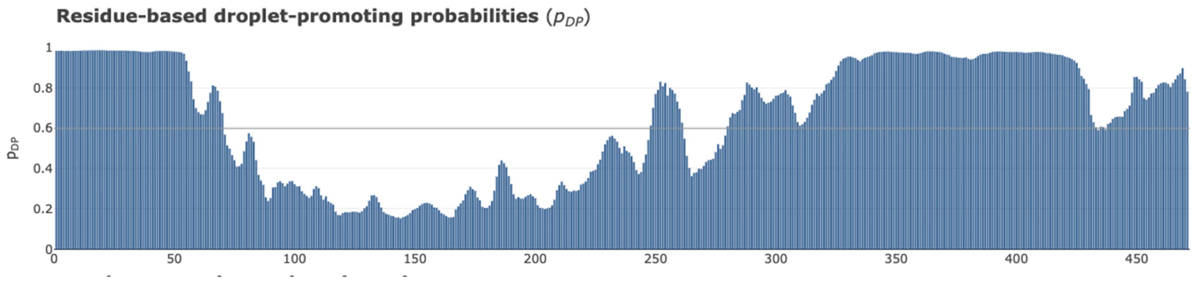

Figure S2 NONO droplet promoting abilities shown as Per residue droplet promoting score (Pdp) as a function of the sequence of NONO. Both N and C-terminal IDRs predicted to be capable of promoting phase separation with a slight dip in Pdp scores on the C-terminal IDR of NONO.

SFPQ276-598 data:

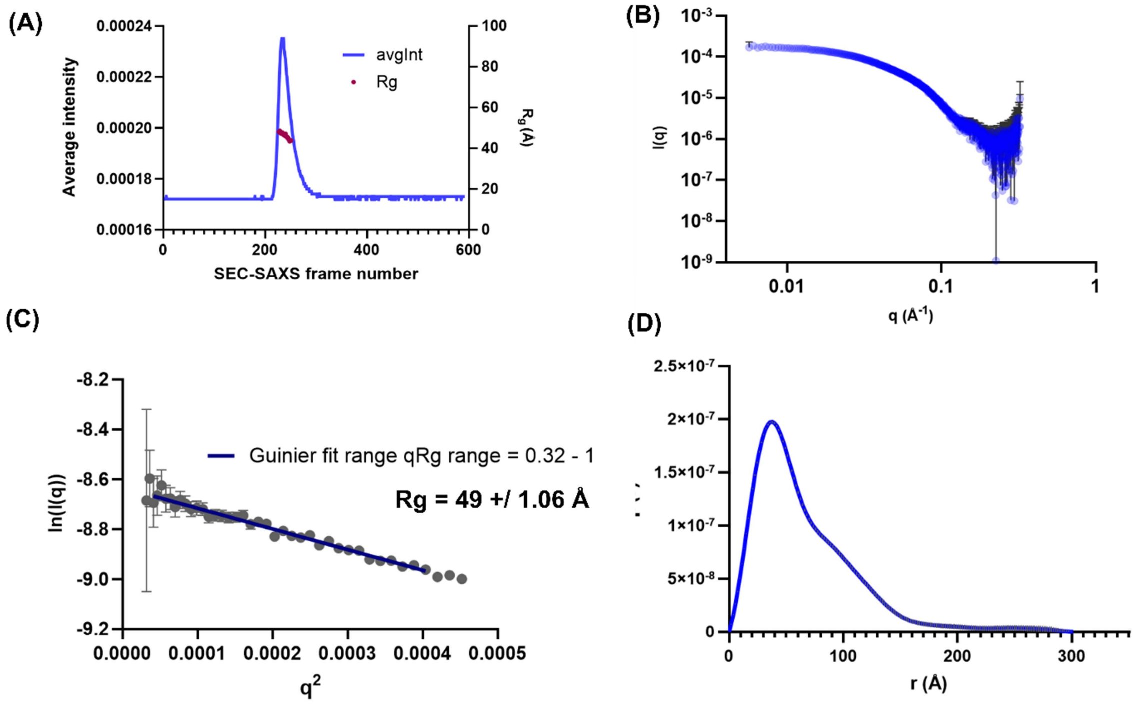

Figure S3 SAXS data on the SFPQ276-598 homodimer: (A) SEC-SAXS CHROMXIS trace indicating X-ray intensity vs. frame number, alongside predicted R_g values (pink). (B) Scattering data shown as a log(I) vs. log(q) plot. (C) Guinier analysis with calculated R_g value. (D) P(r) function for the homodimer.

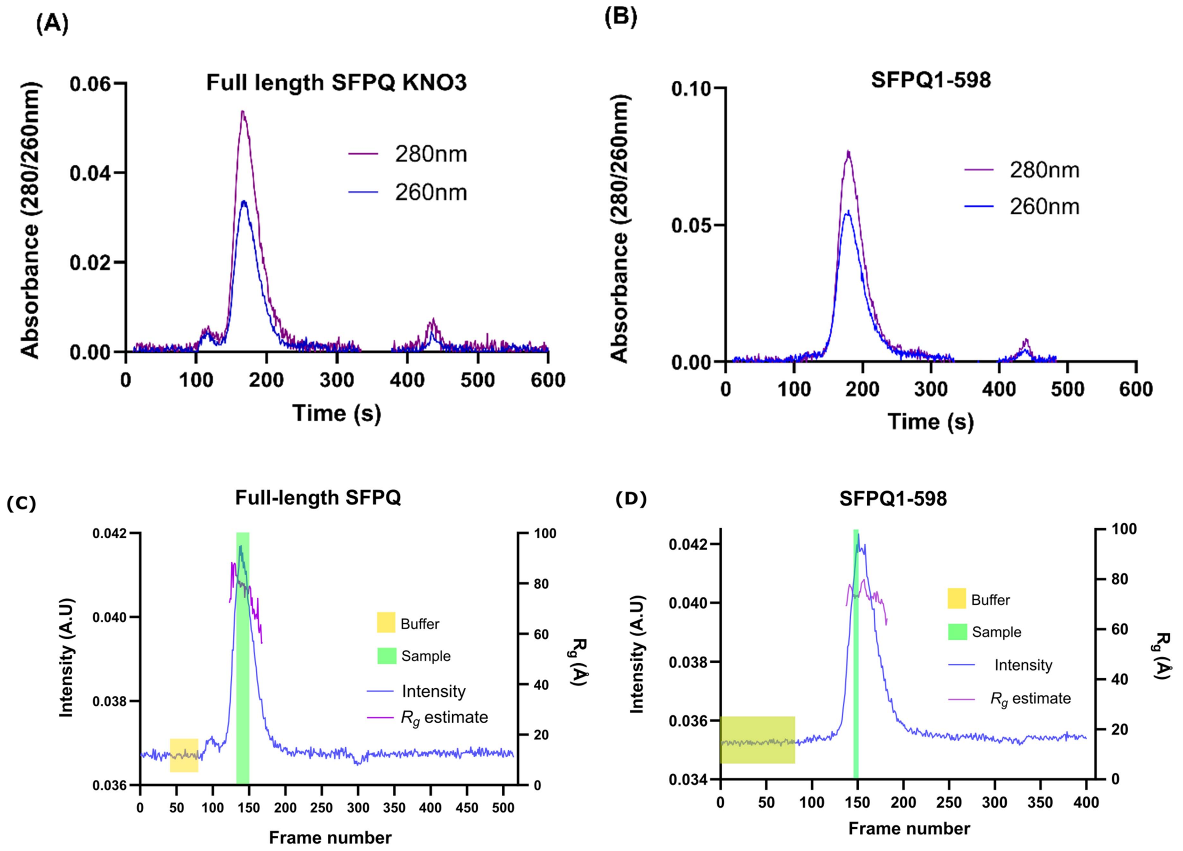

Figure S4 Chromatography traces for SFPQ and SFPQ1-598. (A/B) Chromatography traces indicating absorbance at 260/280nm wavelength against time (seconds) for full-length SFPQ and SFPQ1-598 respectively. (C/D) CHROMIXS traces of full-length SFPQ and SFPQ1-598 with corresponding buffer and sample regions shown in yellow and green respectively. R_g estimates shown as purple traces across the data that have been plotted on the secondary Y-axis.

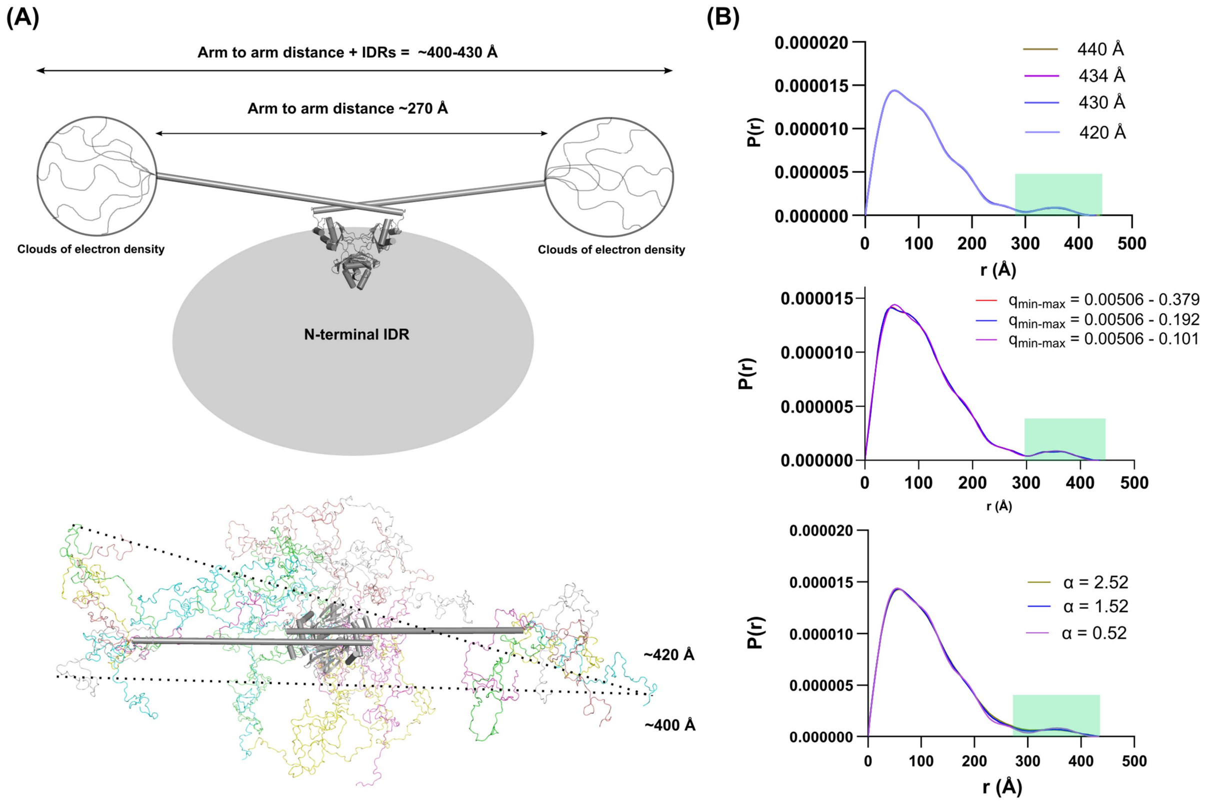

Figure S5 Rationale for the appearance of a distal peak in the P(r) function for full-length SFPQ. (A) Top: A simple model showing that the C-terminal IDRs should act as clouds of electron density constrained at a long distance(~300-434 Å) from one another by the long rigid arms of the dimer core. This should in theory contribute many long-distance pairwise correlations. Bottom: Measurements taken on the EOM ensemble indicate that many measurements across the structure fall within this range. (B) Top: different D_{max} values still allow the peak (highlighted by the green box) to persist in the function. Middle: fitting the regularised curve of the function to different q-ranges in the data still allows the distal peak to persist. Bottom: increasing the GNOM regularisation parameter (α) still shows that the peak largely persists in the data.

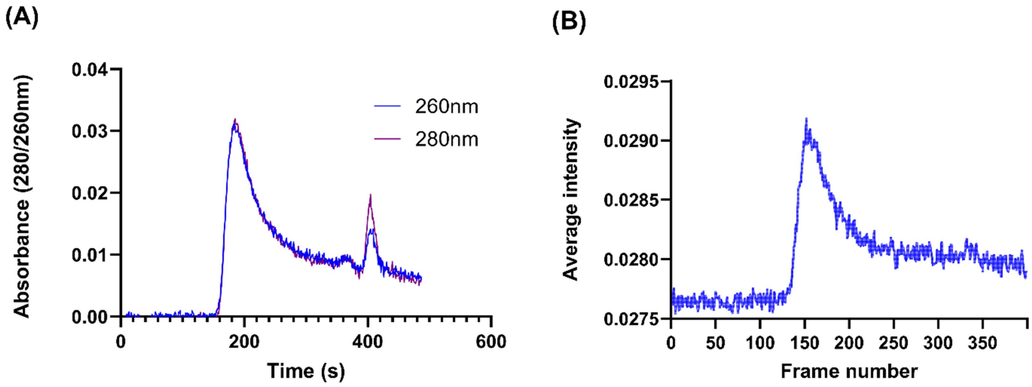

Figure S6 SEC-SAXS chromatography trace for SFPQ1-598 in low salt conditions. (A) Absorbance chromatogram (280/260nm wavelength) of SFPQ1-598 in low salt buffer against time. (B) CHROMIXS trace for the same experiment indicating X-ray intensity against frame number. This dataset suffered from an elevated 260:280nm absorbance ratio likely due to the effects of buffer constituents. Given this sample came from the same batch as the nitrate experiments which had UV absorbance ratios consistent with that of pure protein, and was then dialysed into a low salt buffer it is unlikely that the elevated 260nm readings are the result of contaminating nucleic acid, rather buffer constituents interfering with absorbance ratios.

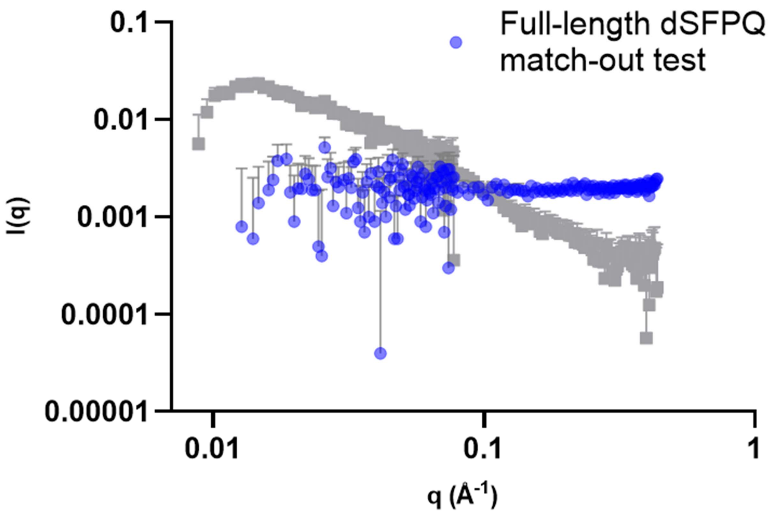

Figure S7 Match-out testing for deuterated full-length SFPQ. Log(I) vs log(q) plot showing the scattering from full-length deuterated SFPQ dialysed to its 95% D_2O match-point (blue). Flat-line scattering indicates that contrast matching was successful. Scattering data on the mixture of dSFPQ and hSFPQ that showed partner exchange also plotted here as a reference (grey).

Table S1 Cancer-associated cysteine mutations in the C-terminal IDR of DBHS proteins. Mutations were pooled from the COSMIC database.

Gene	COSMIC AC	COSMIC ID	Confirmed somatic?	HGVS.c	HGVS.p	Equivalent SFPQ position:	Primary histology	Tissue distribution:
NONO	1	COSV52137725	Yes	c.1366C>T	R456C	Close to end of C-terminal IDR	Adenocarcinoma	Large intestine
NONO	1	COSV99339020	Yes	c.1402C>T	R468C	End of C-terminal IDR	Ductal carcinoma, adenocarcinoma	Pancreas, prostate
NONO	1	COSV52139352	Yes	c.1405C>T	R469C	End C-terminal IDR	Intestinal adenocarcinoma	Stomach
SFPQ	1	COSV100599533	Yes	c.1921G>T	G641C	Middle of C-terminal IDR	Carcinoma	Lung
SFPQ	1	COSV61759221	No	c.1939G>T	G647C	Middle of C-terminal IDR	Carcinoma	Lung
PSPC1	1	COSV100142669	Yes	c.1426G>T	G476C	Middle/End of C-terminal IDR	Carcinoma	Aerodigesitve tract
PSPC1	1	COSV58886423	No	c.1495A>T	S499C	Towards end of C-terminal IDR	Carcinoma	Lung
PSPC1	1	COSV107443250	Yes	c.1510G>T	G504C	End of C-terminal IDR	Carcinoma	Lung
PSPC1	1	COSV58891636	Yes	c.1558C>T	R520C	End of C-terminal IDR	Carcinoma	Biliary tract
